# Identification and characterization of microRNAs in the flag leaf and developing seed of wheat (*Triticum aestivum* L.)

**DOI:** 10.1186/1471-2164-15-289

**Published:** 2014-04-16

**Authors:** Ran Han, Chao Jian, Jinyang Lv, Yan Yan, Qing Chi, Zhanjie Li, Qian Wang, Jin Zhang, Xiangli Liu, Huixian Zhao

**Affiliations:** 1 State Key Laboratory of Crop Stress Biology for Arid Areas, Northwest A & F University, Yangling 712100, Shaanxi, China; 2 College of Life Sciences, Northwest A & F University, Yangling 712100, Shaanxi, China

**Keywords:** MicroRNA, *Triticum aestivum*, Flag leaf, Seed development, Small RNA sequencing, Expression profile, miRNA target

## Abstract

**Background:**

MicroRNAs (miRNAs) regulate various biological processes in plants. Considerable data are available on miRNAs involved in the development of rice, maize and barley. In contrast, little is known about miRNAs and their functions in the development of wheat. In this study, five small RNA (sRNA) libraries from wheat seedlings, flag leaves, and developing seeds were developed and sequenced to identify miRNAs and understand their functions in wheat development.

**Results:**

Twenty-four known miRNAs belonging to 15 miRNA families were identified from 18 MIRNA loci in wheat in the present study, including 15 miRNAs (9 MIRNA loci) first identified in wheat, 13 miRNA families (16 MIRNA loci) being highly conserved and 2 (2 MIRNA loci) moderately conserved. In addition, fifty-five novel miRNAs were also identified. The potential target genes for 15 known miRNAs and 37 novel miRNAs were predicted using strict criteria, and these target genes are involved in a wide range of biological functions. Four of the 15 known miRNA families and 22 of the 55 novel miRNAs were preferentially expressed in the developing seeds with logarithm (log2) of the fold change of 1.0 ~ 7.6, and half of them were seed-specific, suggesting that they participate in regulating wheat seed development and metabolism. From 5 days post-anthesis to 20 days post-anthesis, miR164 and miR160 increased in abundance in the developing seeds, whereas miR169 decreased, suggesting their coordinating functions in the different developmental stages of wheat seed. Moreover, 8 known miRNA families and 28 novel miRNAs exhibited tissue-biased expression in wheat flag leaves, with the logarithm of the fold changes of 0.1 ~ 5.2. The putative targets of these tissue-preferential miRNAs were involved in various metabolism and biological processes, suggesting complexity of the regulatory networks in different tissues. Our data also suggested that wheat flag leaves have more complicated regulatory networks of miRNAs than developing seeds.

**Conclusions:**

Our work identified and characterised wheat miRNAs, their targets and expression patterns. This study is the first to elucidate the regulatory networks of miRNAs involved in wheat flag leaves and developing seeds, and provided a foundation for future studies on specific functions of these miRNAs.

## Background

Wheat (*Triticum aestivum* L.) is a major staple crop for human diet. With the increase in global population, the shortage of food has become increasingly serious. Therefore, improvement of wheat yield is vital for relieving food shortages. Conventional breeding approaches via manipulation of genetic variation have been successful in improving the important agronomic traits of cereals; however, further wheat improvement requires deep understanding of the molecular mechanisms that control wheat growth and development [1].

Small RNAs (sRNAs) have been found to control cellular metabolism, growth and differentiation, to maintain genome integrity, and to combat viruses and mobile genetics elements in eukaryotes [2]. According to the present knowledge, these regulatory sRNAs are classified into microRNAs (miRNAs), short interfering RNAs (siRNAs), and Piwi-interacting RNAs in metazoans. miRNAs are produced from non-coding single-stranded RNA precursors (pri-miRNAs) that are transcribed by RNA polymerase II to generate an imperfect self-complementary, stem-loop secondary structure [3,4]. In higher plants, a pri-miRNA is trimmed and spliced into an sRNA duplex consisting of an miRNA and its complementary sequence (miRNA*) by Dicer-like1 (DCL1) with the help of the dsRNA binding protein HYL1 and the dsRNA methylase HEN1 [5-7]. A mature miRNA, which is typically 20 nt to 22 nt long, is incorporated in the RNA-induced silencing complex to down-regulate the expression of its target genes *in trans* through a base pairing mechanism, whereas miRNA* is assumed to be degraded [5,8]. miRNAs display near-perfect complementarity to their target mRNAs and interfere with target gene expression by mRNA cleavage, which occurs at the 10^th^ and 11^th^ positions of miRNAs, or by inhibition of translation in plants [9,10]. In plants, miRNAs are the second most abundant sRNAs [11], acting as powerful endogenous regulators. For example, many distinct miRNAs target transcripts encoding for various transcription factors that control plant development and phase transition in *Arabidopsis*[12], rice [13] and maize [14], whereas others are involved in biotic and abiotic stress responses [15,16]. The number of annotated miRNAs in miRBase database has exponentially increased in recent years. To date, 7,390 miRNAs have been identified in the plant kingdom, and the information has been deposited in miRBase (Release 20.0, June 2013; http://www.mirbase.org). However, most of these miRNAs have been identified in plants whose whole genome sequences are available; some of these plants include *Oryza sativa* (713), *Populus trichocarpa* (401), *Arabidopsis thaliana* (337), *Brachypodium distachyon* (464) and *Zea mays* (321). Similar to many gene regulatory systems, miRNAs are both conserved and diverse among plant lineages. Some miRNAs are conserved in angiosperms or even in embryophyta [16], whereas others are species specific, reflecting their fast-evolving and functionally diverging nature [16,17]. Recent studies have investigated the functions of miRNAs in the seed development of various plants, such as *Arabidopsis*[18,19], rice [13,20,21], maize [22], barley [23] and wheat [24]. Plants expressing the miR160-resistant auxin response factor 17 (ARF17) may cause abnormal embryo symmetry [18]. miR164 regulates NAC-domain target genes in *Arabidopsis*, and perturbation of miR164-directed regulation causes developmental abnormalities in embryonic, vegetative and floral organs [12]. miRNAs are involved in many regulatory pathways that control seed development in *Arabidopsis*; therefore, loss-of-function miRNA mutations may lead to developmental defects or death [19]. miR156 targets squamosa promoter-binding protein-like 10 (SPL10) and SPL11, and the regulation of these targets prevents premature gene expression during early embryogenesis [19]. In barley, it was found that the regulatory functions of miRNAs peak during the transition between the pre-storage and storage phases in seed development; miRNAs regulate the development of cereal grain through phytohormone response pathways [23]. A recent study has found that miR397 overexpression improves rice yield by increasing grain size and promoting panicle branching [21]. Therefore, understanding the involvement of miRNAs in plant development is necessary to elucidate their functions in seed development and to promote yield improvement not only in rice but also in other cereal crops by manipulating related miRNA genes.

To date, less than 220 miRNAs have been identified in wheat, 42 of which have been registered in the miRBase/*Triticum aestivum* (Release 20.0, June 2013; http://www.mirbase.org) [25-29]. Considerable data are available on the miRNAs involved in the development of rice, maize and barley. In contrast, little is known about the miRNAs and their regulatory functions in the development of wheat. Therefore, identification and functional analysis of miRNAs in wheat are urgently needed.

Wheat development is a complex event wherein the expansion and specialisation of different tissues are controlled by complicated interactions of signalling and gene regulation networks. The complex developmental events crucial to the development of a mature grain composed of the embryo, endosperm (starchy endosperm and aleurone) and outside layer (seed coat and pericarp) take approximately 40 days-post-anthesis (DPA). Grain development can be divided into three stages based on morphological changes and metabolite accumulation: pre-storage, storage (or maturation) and desiccation [30,31]. The pre-storage phase corresponding to the first 5 DPA is featured by extensive mitotic activity in both embryo and endosperm. The transition to storage phase, characterised by dramatic transcriptional changes, occurs at 5 DPA to 10 DPA; this transition initiates the differentiation of tissues that will constitute the mature grain [30]. Throughout the storage phase, which lasts up to approximately 25 DPA, aleurone and embryonic tissues acquire desiccation tolerance, whereas endosperm cells undergo endoreduplication and accumulate storage metabolites (mainly starch and proteins) [31]. Flag leaf is essential in wheat reproduction development because it contributes approximately 45% to 58% of the total photosynthetic activity [32] and approximately 41% to 43% of the carbohydrates for grain filling [33].

Our long-term goal is to reveal the roles of miRNAs in wheat development. The objective of this study is to systematically identify miRNA species in wheat tissues during different developmental stages. To achieve this, we sequenced five sRNA libraries from wheat seedlings, flag leaves and immature seeds at 5 DPA (5-d seed), 10 DPA (10-d seed) and 20 DPA (20-d seed), respectively, to identify the miRNAs and understand their functions in wheat flag leaf and seed development. We identified known and novel miRNAs based on the presence of their precursors in wheat EST databases or wheat genome shotgun-sequence assemblies and their abundance as well as based on the detection of miRNA*. We also predicated the potential targets for the identified miRNAs and then analysed the expression of their profiles. This study is the first to provide useful information for uncovering the regulatory networks of miRNAs in wheat flag leaves and developing seeds.

## Results and discussion

### Diverse sRNA population in different wheat tissues or developmental stages

All sequencing data were first processed by filtering adaptor sequences and removing low-quality reads, and reads with sequences larger than 30 nt and smaller than 18 nt using the SOAPnuke software (http://soap.genomics.org.cn/) developed by BGIA, and clean reads were generated for each sRNA library. Identical reads were subsequently pooled to create a list of non-redundant sequences (unique sequences). A total of 74,590,133 clean reads representing 19,872,325 unique reads 18 nt to 30 nt long were obtained from deep sequencing of the five sRNA libraries (Table 1). The number of unique reads increased in flag leaves and developing seeds of 5-d, 10-d and 20-d. This observation is consistent with a previous study, which found the increasing unique signatures in developing barley grain samples at 1 DPA to 5 DPA, 6 DPA to 10 DPA, and 11 DPA to 15 DPA [23]. These data suggest that wheat developing seeds have a more complex sRNA population than vegetative tissues.

**Table 1:** Read statistics in five sRNA libraries

**Tissue**	**Redundant**		**Unique**	
	**Clean read**^**a**^	**Wheat genome-matched read**^**b**^	**Clean read**^**a**^	**Wheat genome-matched read**^**b**^
Seedlings^c^	13,931,738	6,344,991 (45.54%)	3,670,844	1,947,422 (53.05%)
Flag leaves^d^	13,595,341	7,499,877 (55.17%)	3,089,194	1,823,512 (59.03%)
5-d seeds	14,569,411	8,436,803 (57.91%)	3,214,958	1,619,493 (50.37%)
10-d seeds	14,181,881	8,203,547 (57.85%)	3,857,235	2,114,232 (54.81%)
20-d seeds	18,311,762	10,833,109 (59.16%)	6,040,094	3,515,176 (58.20%)
Total	74,590,133	41,352,236 (55.39%)	19,872,325	11,028,735 (55.45%)

^a^ 18 nt to 30 nt in length.

^b^ Referring to clean reads perfectly matched with wheat genome shotgun-sequence assemblies (http://mips.helmholtz-muenchen.de/plant/wheat/uk454survey/index.jsp).

^c^ Seedlings after vernalisation in the field (at five-leaf stage).

^d^ From heading wheat plants.

Only 41,352,236 of the 74,590,133 redundant reads (55.39%) and 11,028,735 of the 19,872,325 unique reads (55.45%) were matched perfectly to wheat genome shotgun-sequence assemblies (http://mips.helmholtz-muenchen.de/plant/wheat/uk454survey/index.jsp) (Table 1). The summary of sRNA sequencing in the five libraries or tissues is listed in Additional file 1.

**Figure 1:**
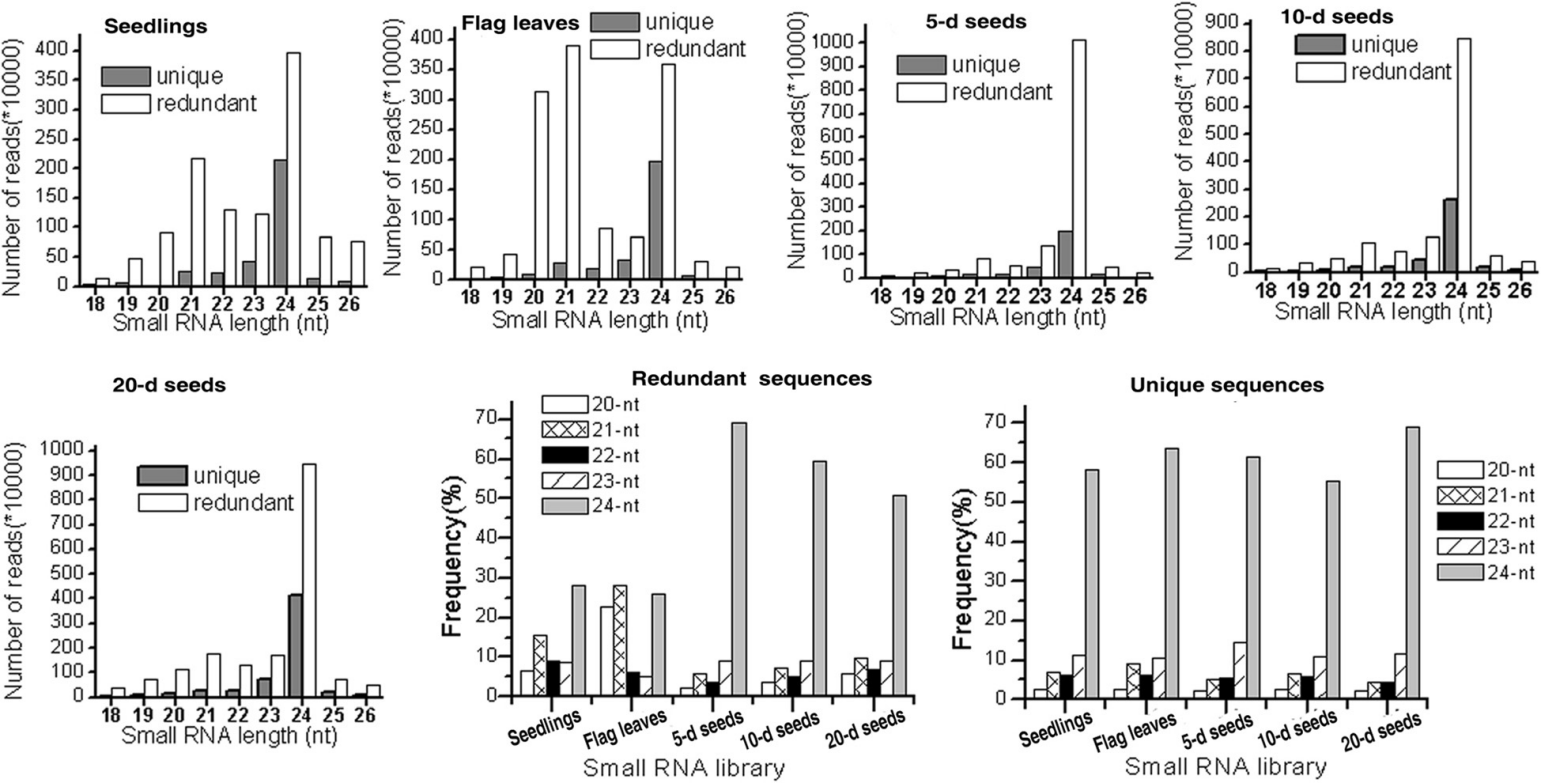
**Size distribution of redundant and unique short RNA sequences.** Number of redundant and unique small RNA (sRNA) sequences from seedlings, flag leaves and immature seeds at 5 (5-d seeds), 10 (10-d seeds), and 20 (20-d seeds) days post-anthesis are shown separately. The frequency of redundant and unique 20 to 24 nt sRNAs in the seedlings, flag leaves and 5-d, 10-d and 20-d seeds are also shown, respectively. The frequency is expressed as a percentage of the total number of clean reads for each tissue.

We focused on the distribution of 18 nt to 26 nt reads in the five libraries or tissues because most of the sRNAs with known functions are 20 nt to 24 nt long. The size distribution patterns of the sRNA reads in the five wheat tissues suggest a distinct sRNA population in a particular wheat tissue or developmental stage (Figure 1). In the seedlings, the most abundant sRNA reads were 24 nt long (about 30%), followed by reads of 21 nt long (15%), the typical length of canonical miRNAs (Figure 1). These results are consistent with previous findings in *Arabidopsis*[34], rice [13], soybean [35] and apple [36]. However, sRNA reads from flag leaves were characterised with 24, 21 and 20 nt (27%, 30% and 22%, respectively) as the major size classes (Figures 1). The distribution of sRNA reads from flag leaves is similar to what has been observed in a previous study [37]. sRNAs with different sizes perform different functions. For example, post-transcriptional gene silencing is usually mediated by 21 nt sRNAs, whereas gene silencing mediated by RNA-dependent DNA methylation and heterochromatin maintenance are usually performed by 24 nt sRNAs [38,39].

The sRNA reads from the 5-d, 10-d and 20-d seeds shared similar size distribution patterns, with 24 nt as the dominant class of sRNAs (Figure 1). The frequencies of 24 reads decreased from the 5-d seeds to the 20-d seeds, while 21 nt increased. This result is in agreement with previous data on the early development of barley seed samples at 1 DPA to 5 DPA, 6 DPA to 10 DPA, and 11 DPA to 15 DPA [23]. However, if unique sequences are considered, 24 nt reads composed the predominant class of sRNAs (approximately 55% to 70%) in all the five libraries or tissues, as compared with the 20 to 23 nt reads (3% to 15%) (Figure 1). The five sRNA libraries shared less than 1.2% of the 21 nt sRNAs and less than 0.4% of the 24 nt sRNAs, indicating diverse sRNA populations in the tested tissues. Overall, these results indicate that distinct pools of sRNA work in different wheat tissues or developmental stages.

### Characterization of highly to moderately conserved known miRNAs

In this study, sRNA reads with sequence similarity above 90% to rRNAs, tRNAs, snRNAs and snoRNAs were removed by BLASTN alignment against the data deposited in Rfam 10.0 (http://rfam.janelia.org/), and unique sRNA sequences perfectly matched to wheat genome shotgun-sequence assemblies were aligned to known plant miRNAs in miRBase (Release 20.0, http://www.mirbase.org). As a result, 24 known miRNAs representing 15 miRNA families were identified from 18 MIRNA loci in wheat (Table 2 and Additional file 2). Of these 24 known miRNAs, 9 (9 MIRNA loci) have been registered in miRBase/*Triticum aestivum* (Release 20.0, http://www.mirbase.org), and the remaining 15 (9 MIRNA loci) were first identified in wheat in the present study (Additional files 2, 3 and 4). Among the 15 known miRNAs (from 9 MIRNA loci in wheat), 6 pairs are miRNA/miRNA*. In the present study, these known miRNAs/families were sorted into two categories: highly conserved miRNAs and moderately conserved miRNAs. The former miRNAs are conserved in all three dicots (*Arabidopsis*, soybean and *Populus*) and three monocots (rice, maize and *Brachypodium*) whose genome sequences are available; the latter miRNAs are only conserved in some of these plant species, but not in the all six plant species described above (miRBase 20.0). Of the 15 known miRNA families (18 MIRNA loci), 13 (16 MIRNA loci) are highly conserved and 2 (2 MIRNA loci) are moderately conserved (Table 2 and Additional file 2).

**Table 2:** Known miRNAs identified in the five wheat sRNA libraries or tissues

**miRNA family #**	**Number of miRNA species**	**Abundance (RPM)**^**☆**^						**Logarithm of the fold change**^**★**^			
		**Seedlings**	**Flag leaves**	**5-d seeds**	**10-d seeds**	**20-d seeds**	**Total**	**Flag leaves**	**5-d seeds**	**10-d seeds**	**20-d seeds**
Highly conserved											
miR156	2	15055	36735	1193	4048	4804	61835	1.3	−3.7	−1.9	−1.6
miR159	1	17	82	13	4	11	127	2.3	−0.4	−2.1	−0.6
miR160	1	1	2	1	0	8	12	1.0	0.0	-	3.0
miR164	1	206	145	135	246	244	976	−0.5	−0.6	0.3	0.2
miR166^※^	2	2345	2782	4556	1239	2350	13272	0.2	1.0	−0.9	0.0
miR167	3	3678	9319	1130	2213	4623	20963	1.3	−1.7	−0.7	0.3
miR168^※^	2	56,845	134,020	15,007	18,239	14,070	238182	1.2	−1.9	−1.6	−2.0
miR169	1	24	1	87	68	19	199	−4.6	1.9	1.5	−0.3
miR171	1	26	17	18	9	7	77	−0.6	−0.5	−1.5	−1.9
miR172^※^	2	422	2273	47	89	102	2933	2.4	−3.2	−2.2	−2.0
miR395	1	5	0	0	0	0	5	-	-	-	-
miR396^※^	4	303	422	91	65	40	921	0.5	−1.7	−2.2	−2.9
miR398	1	1	15	0	0	0	16	3.9	-	-	-
Moderately conserved											
miR1122	1	1	1	0	0	0	2	0.0	-	-	-
miR1318^※^	1	10	366	1	1	1	379	5.2	−3.3	−3.3	−3.3

^**#**^ Highly conserved refers to miRNAs that are conserved in all three dicots (*Arabidopsis*, soybean and *Populus*) and three monocots (rice, maize and *Brachypodium*) whose genome sequences are available. Moderately conserved refers to miRNAs that only conserved in some of these plant species, but not in all the six plant species described above (miRBase 20.0, June 2013; http://www.mirbase.org).

^※^Represents miRNA families that were first detected in wheat in this study.

^☆^Abundance reflected by normalised reads (reads per million of total miRNA reads, RPM).

^★^Logarithm of the fold change was calculated using log2, whereas the fold change is the ration of the abundance of a miRNA family in flag leave, 5-d seeds, 10-d seeds or 20-d seeds to the abundance of the same miRNA family in seedlings). "-" refers to no data.

The abundance of these known miRNAs, as reflected in normalised reads (reads per million of total miRNA reads, RPM), was further compared in this study. The highly conserved miRNAs or families showed significant variation among the families in all the five tissues (Table 2 and Additional file 2). The highest read abundance (approximately 238,000 RPM) was detected in the miR168 family and was 3.8 to 78 times more abundant than the other miRNA families, including miR156, miR166, miR167 and miR172, whose abundance ranged from about 2,900 RPM to 62,000 RPM (Table 2). Although low expression (976 RPM and 921 RPM, respectively) was observed for both miR164 and miR396 families, their expression level was still about 4 to 200 times greater than any of the 6 remaining highly conserved miRNA families (Table 2 and Additional file 2). Similarly, the two moderately conserved miRNA families exhibited great variation in abundance between the families (Table 2). Variation in expression levels of different miRNA species was also found in other plant species, such as grapevine [40] and apple [36].

### Novel miRNA species in wheat

sRNA reads with no sequence similarity to known miRNAs were further analyzed for potential new miRNA species. These unique sequences with 20 to 23 nt were analyzed against wheat EST database (http://www.ncbi.nlm.nih.gov/nucest/?term=wheat) or wheat genome shotgun-sequence assemblies (http://mips.helmholtz-muenchen.de/plant/wheat/uk454survey/index.jsp). The EST sequences or the contigs from the wheat genome shotgun-sequence assemblies that matched to the sRNA reads were extracted from the databases and analyzed for the existence of pre-miRNA sequences that can form a hairpin secondary structure using miRNA prediction software MIREAP (http://sourceforge.net/projects/mireap/), the potential new miRNAs predicted meeting the common criteria (see details in Methods). Then, novel miRNAs were further identified based on both their abundance (at least 5 RPM in at least one of the five tissues examined) and the detection of miRNA*s, because accumulations of miRNA*s are strong supporting evidence for the cleavage by DCLs to produce functional mature miRNAs during miRNA biogenesis in plants [5]. A total of 55 novel miRNAs corresponding to 54 precursors which can form qualified secondary structures were identified in the present study (Table 3, Additional file 5), and of which, 6 mature miRNA sequences were previously described in wheat [24,26]. The detailed information of all the 55 novel miRNAs, including the pre-miRNA sequences and the structures, the loci of the mature miRNA sequences and their miRNA*s, and the alignments of all novel miRNAs to their precursor sequences, were shown in Additional files 6 and 7. While, 53 miRNAs with abundance more than 5 RPM in at least one of the five tissues tested but without miRNA*s detected were identified as candidate miRNAs (Additional file 8), their loci, pre-miRNA sequences and structures, and reads in deep sequencing were also list in Additional files 6 and 7. A recent review [41] has reported that 65.5% of the known miRNAs in wheat begin with a 5′ uridine and that 56.5% are 21 nt long; these properties are typical in miRNAs from other species [42,43]. In the present study, 56.4% (31/55) of the novel miRNAs started with a U at their 5′-end, and that 65.5% (36/55) are 21 nt long (Additional file 5). As been found in *Arabidopsis* and apple, new species-specific miRNAs are young miRNAs that have recently evolved and are often expressed at a lower level than conserved miRNAs [34,36,44]. This observation is also true for most of the 55 novel miRNAs identified in the present study (Table 3, Additional file 5).

**Table 3:** Summary of newly identified 55 novel miRNAs in the five wheat libraries or tissues

**miRNA**	**Mature sequence(5′→3′)**^**☆**^	**Length (nt)**	**Abundance (RPM)**^**§**^		
			**Seedlings**	**Flag leaves**	Developing seeds*
tae-miR1120b	UUCUUAUAUUGUGGGACAGAG	21	26	131	56
tae-miR1120c	UAAUAUAAGAACGUUUUUGAC	21	0	24	0
tae-miR1122b	AGACUUAUAUGUAGGAACGGA	21	0	0	10
tae-miR1122c	UCUAAUAUUAUGGGACGGAGG	21	4	8	4
tae-miR1127b	ACAAGUAUUUCUGGACGGAGG	21	0	0	17
tae-miR1130b	UCUUAUAUUAUGGGACGGAGG	21	0	10	0
tae-miR1137b	UCCGUUCCAGAAUAGAUGACC	21	9	13	28
tae-miR167c	UGAAGCUGCCAGCAUGAUCUGC	22	67	173	88
tae-miR1847	ACCUGCAGUUGGGCCAAUGAC	21	47	106	20
tae-miR2275	UUUGGUUUCCUCCAAUAUCUCG	22	0	0	12
tae-miR396	AACUGUGAACUCGCGGGGAUG	21	6	11	35
tae-miR397	UCACCGGCGCUGCACACAAUG	21	2	92	5
tae-miR5048	UUUGCAGGUUUUAGGUCUAAGU	22	1,142	1,274	0
tae-miR5049	**AAUAUGGAUCGGAGGGAGUAC**	21	1	13	1
tae-miR5062	UGAACCUUAGGGAACAGCCGCAU	23	510	1,509	2,932
tae-miR5175	UUCCAAAUUACUCGUCGUGGU	21	0	129	34
tae-miR5384	UGAGCGCGCCGCCGUCGAAUG	21	0	12	0
tae-miR6197	**UCUGUAAACAAAUGUAGGACG**	21	29	71	131
tae-miR7757	AUAAAACCUUCAGCUAUCCAUC	22	67	83	78
tae-miR9652-3P	AAGCUUAAUGAGAACAUGUG	20	0	14	1
tae-miR9652-5P	CCUGUUUGUCAUUAAGUUUCUU	22	2	0	10
tae-miR9653	UUUGAGACUUUGGCCAUGGCC	21	0	0	15
tae-miR9654a	UUCUGAAAGGCUUGAAGCGAAU	22	0	0	135
tae-miR9654b	UUCCGAAAGGCUUGAAGCGAAU	22	1	3	34
tae-miR9655	CAAGGGAAGGAAGUAGCCAAC	21	15	1	1047
tae-miR9656	CUUCGAGACUCUGAACAGCGG	21	0	0	18
tae-miR9657a	UGUGCUUCCUCGUCGAACGGU	21	0	0	46
tae-miR9657b	UUCGUCGGAGAAGCAUGUUGC	21	0	0	60
tae-miR9657c	**CGUGCUUCCUCGUCGAACGGU**	21	16	39	29
tae-miR9658	AUCGUUCUGGGUGAAUAGGCC	21	7	10	299
tae-miR9659	UCCAAUGGUUGUUCACGGCAUC	22	0	0	248
tae-miR9660	UUGCGAGCAACGGAUGAAUC	20	0	0	21
tae-miR9661	UGAAGUAGAGCAGGGACCUCA	21	2	1	27
tae-miR9662a	UUGAACAUCCCAGAGCCACCG	21	402	488	898
tae-miR9662b	UGAACAUCCCAGAGCCACCGG	21	0	488	821
tae-miR9663	AAGCGUAGUCGAACGAAUCUG	21	1,634	5,562	10,441
tae-miR9664	UUGCAGUCCUCGAUGUCGUAG	21	243	305	1122
tae-miR9665	GCUAGCAGUGUAAACUCAAAUCA	23	0	0	9
tae-miR9666a	CGGUAGGGCUGUAUGAUGGCGA	22	46	47	1,519
tae-miR9666b	CGGUUGGGCUGUAUGAUGGCGA	22	8,913	29	477
tae-miR9666c	GCCAUCAUACGUCCAACCGUG	21	10	0	0
tae-miR9667	AAAUAUGGCAAACAAUGAAUG	21	0	0	27
tae-miR9668	CCAAUGACAAGUAUUUUCGGA	21	0	10	9
tae-miR9669	UACUGUGGGCACUUAUUUGAC	21	9	0	0
tae-miR9670	AGGUGGAAUACUUGAAGAAGA	21	140	218	409
tae-miR9671	UGACUUUACACAACUGUCCGGC	22	6	13	0
tae-miR9672	CCACGACUGUCAUUAAGCAUC	21	92	366	36
tae-miR9673	UAAGAAGCAAAUAGCACAUG	20	4	14	11
tae-miR9674a	GCAUCAUCCAUCCUACCAUUC	21	143	346	337
tae-miR9674b	AUAGCAUCAUCCAUCCUACCC	21	362	453	817
tae-miR9675	UUUAUGAUCACUCUCGUUUUG	21	0	32	0
tae-miR9676	UGGAUGUCAUCGUGGCCGUACA	22	57	63	19
tae-miR9677	**UGGCCGUUGGUAGAGUAGGAGA**	22	1	9	344
tae-miR9678	**UCUGGCGAGGGACAUACACUGU**	22	4	0	411
tae-miR9679	**CAGAACCAGAAUGAGUAGCUC**	21	15	32	59

^☆^The mature sequence bold were previously described in wheat (Wei et al., 2009 [26]; Meng et al., 2013 [24]).

^§^Abundance reflected by normalised reads (reads per million of total miRNA reads, RPM).

*Including 5-d seeds, 10-d seeds, and 20-d seeds, thereby the abundance referring to the total of abundance of each miRNA in the three stages of developing seeds.

### Target gene prediction for novel miRNAs

To understand the functions of the novel miRNAs identified, the putative targets of these miRNAs were predicted by using a web-based psRNA Target program (http://plantgrn.noble.org/psRNATarget/) with default parameters given in the Methods section. Thirty-seven of the 55 novel miRNAs (67.3%) had predicted targets that met the criteria (Additional file 9). According to the data in miRBase, 34 of the 42 (80.9%) wheat miRNAs have predicted targets, which is a little more than our result. As shown in Additional file 9, the predicted targets included transcript factors, protein kinases, enzymes, cellular components, receptors and transporters involved in multiple cellular processes, indicating the extensive functions of miRNAs in gene regulation networks. For example, seed-specific tae-miR1127b which were only present in developing seed (Additional file 5) targeted riboflavin biosynthesis protein Rib gene/amino acid permease gene, which function in seed development [45,46]. This result is consistent with its functions reported earlier. Seed-specific tae-miR9653 and tae-miR9661 targeted Zinc finger transcription factor-like protein and F-box domain containing protein, respectively (Additional file 9). These genes have important functions throughout plant development [47,48].

### Preferential expression of different miRNA species in specific wheat tissues

Knowledge about the temporal and spatial expression of miRNAs might provide clues on where these miRNAs function. In the present study, the availability of the five sRNA datasets from the seedlings, flag leaves, 5-d seeds, 10-d seeds and 20-d seeds of wheat provided an opportunity to compare the expression profiles of these miRNAs in different tissues and developmental stages. The datasets from Solexa sequencing indicated that the majority of the miRNAs including the known and the novel miRNAs showed different levels of tissue-biased expression with logarithm (log2) of the fold changes of −4.6 ~ 5.2 for the known miRNAs and −8.6 ~ 7.6 for the novel miRNAs (Table 2 and Additional file 5), whereas the fold change was the ratio of abundance of a miRNA or family in flag leaves, 5-d seeds, 10-d seeds or 20-d seeds, to abundance of the same miRNA or family in seedlings.

**Figure 2:**
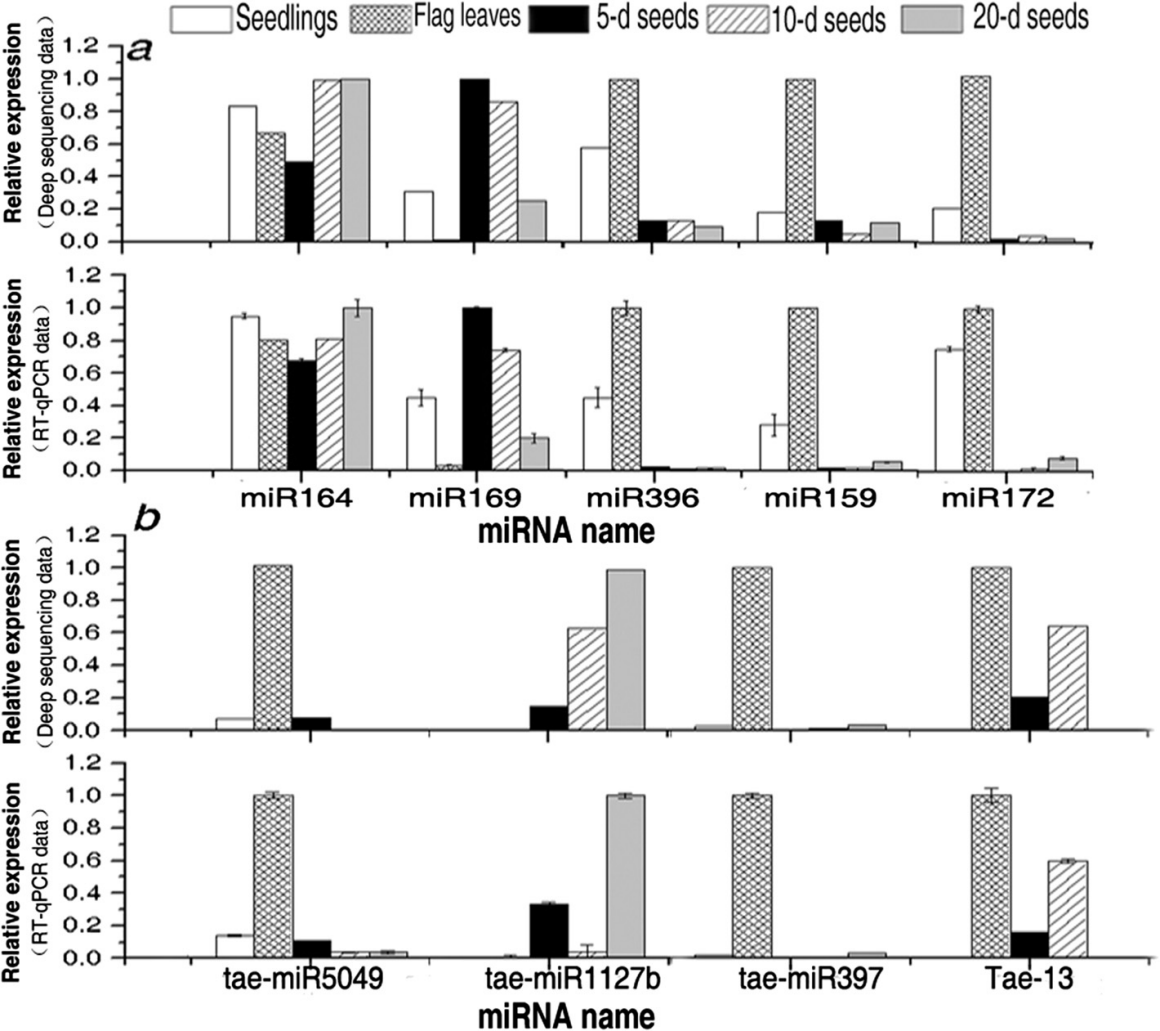
**Comparison of the miRNA expression profiles determined by quantitative real-time RT-PCR (qPCR) and deep sequencing. *a***, known miRNAs; ***b***, novel miRNAs and candidate miRNAs. In qPCR, *UBQ* was used as the internal reference gene, and the relative expression of each miRNA was calculated using a comparative C_T_ (ΔΔC_T_) method. The miRNA sample with the lowest C_T_ value that corresponds to the highest expression level was selected as the calibrator, in which the expression level was set as 1.0. The relative expression levels of the same miRNA in the other four samples were then normalised by comparing with the highest one in the tested tissues. Three independent biological replicates were performed in this experiment. For each sample, qPCR was performed in triplicate. Each column represents the mean of three samples, and error bars represent the standard deviation. In deep sequencing technology, read counts for each miRNA in one sample were normalised to reads per million of total miRNA reads (RPM). The relative expression of each miRNA was calculated by setting the highest RPM of each miRNA across the five samples as 1.0, and the relative expression of the same miRNA in the other four samples was its RPM divided by the highest RPM.

The expression levels of randomly selected representative miRNA species in the five tissues were further determined through quantitative real-time RT-PCR (qPCR) using *UBQ* as the internal reference gene. The *UBQ* gene was confirmed to be relatively stable in the tested wheat tissues (data not shown). Results showed that the relative abundance of almost all the miRNAs determined by qPCR followed similar trends as the read numbers in the libraries (Figure 2). However, we also observed a discrepancy between the qPCR and the sequencing data for novel miRNA tae-miR1127b (Figure 2b), which was expressed at extremely low level in the tissues tested (Additional file 5). This difference might be ascribed to biases introduced to the very low abundant miRNA in some RNA samples when polyadenylation and reverse-transcription were performed with poly (T) adapters into cDNAs using an miRNA cDNA synthesis kit (Takara, Inc., Dalian, China). Thus, the miRNAs’ cDNAs used for qPCR contained biases. Biases could have also been introduced in the sequences of some samples during library generation or sequencing. A contradiction between *in vivo* RNA levels and sequencing results for miRNAs was also reported in grapevine [40] and apple [36]. Further analysis showed that a very significant correlation exists between the sequencing and the qPCR data (Pearson coefficient R^2^ = 0.892, P < 0.01) (Additional file 10), suggesting that the sequencing results are reliable.

**Figure 3:**
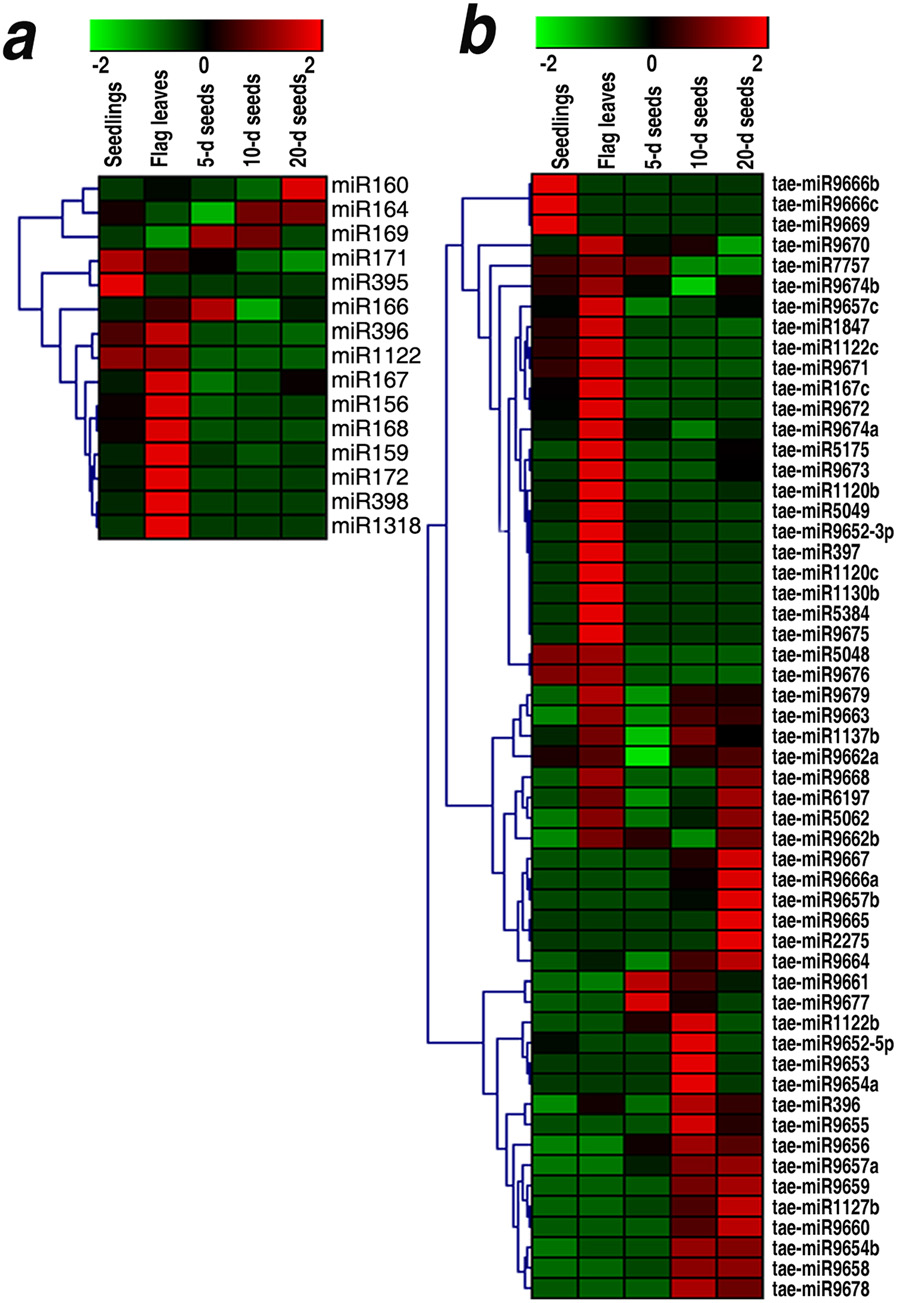
**Expression patterns of the known and the novel miRNAs based on deep-sequencing datasets. *a***, Known miRNAs; ***b***, Novel miRNAs. The bars represent the scale of the relative expression levels of miRNAs (MEAN centred).

Therefore, the expression patterns of all the known and the novel miRNAs identified were profiled based on their sequencing results. Most of the known and the novel miRNAs showed various degrees of tissue-biased expression in the flag leaves or developing seeds, with the logarithm of the fold changes between 0.1 and 7.6 (Figure 3, Table 2 and Additional file 5). Of the 15 known miRNA families, 4 (miR169, miR166, miR164 and miR160) were preferentially expressed in the developing seeds (with the logarithm of the fold changes of 0.3 ~ 3.0 in the developing seeds, more than those in the flag leaves) (Figure 3a, Table 2). From 5 DPA to 20 DPA, miR164 and miR160 increased in abundance, whereas miR169 decreased (Figure 3a, Table 2). This result suggests that these miRNAs might have coordinating functions in different developmental stages of wheat seed. The potential targets of these known miRNAs were predicted (Additional file 11). The expression patterns of these miRNAs were closely related to their functions. miR160 and miR164 targeted Auxin response factor (ARF) and NAC transcription factor (NAC), respectively (Additional file 11), which control key steps in plant development. Auxin is crucial in seed development processes, including pattern formation, cell division and cell expansion [49]. Plants expressing the miR160-resistant ARF17 may cause abnormal embryo symmetry [18]. NAC genes have important functions in developmental processes, such as auxin signalling and defence [50,51]. A recent research has revealed that NAC genes that regulate senescence improve protein, zinc, and iron contents in wheat grain [52]. In the present study, the expression level of miR164 increased with wheat grain development, from 135 RPM in the 5-d seeds to more than 240 RPM in the 10-d and 20-d seeds (Table 2). This result is consistent with the previously reported functions of miR164. miR169 targets a CCAAT-box transcription factor, which is involved in diverse processes, such as embryo development, flowering time control and root development [53]. The decreased abundance of miR169 from the 5-d seeds to the 20-d seeds was coordinated with its functions in seed development. Of the 55 novel miRNAs, 22 showed preferential expression in the different developmental stages of wheat seed (Figure 3b), with the logarithm of the fold change of 1.0 ~ 7.6, and half of these miRNAs (tae-miR1122b, tae-miR9653, tae-miR9654a, tae-miR9656, tae-miR9657a, tae-miR9659, tae-miR2275, tae-miR9665, tae-miR1127b, tae-miR9660, tae-miR9657b and tae-miR9667) were seed specific (Figure 3b, Additional file 5). This result suggests that these novel miRNAs might participate in regulating wheat seed development and metabolism. The predicted targets for these seed-specific miRNAs included transcript factors, enzymes, nucleosome/chromatin assembly factor, ribosome recycling factor and cellular components (Additional file 9), indicating the extensive functions of miRNAs in wheat seed development. For examples, tae-miR9657a, with an increasing abundance from the 5-d seeds to the 20-d seeds, targets a nucleosome/chromatin assembly factor which is essential for DNA replication during cell proliferation. tae-miR1127b which increased in abundance from the 5-d seeds to the 20-d seeds targets an amino acid permease gene, which is important for early seed development and plays a crucial for the uptake of amino acids into the endosperm and supplying amino acids for the developing embryo during early embryogenesis [45]. While, tae-miR9661, with a decreasing expression pattern from the 5-d seeds to the 20-d seeds, targets a F-box domain containing protein in wheat (Additional files 3 and 5). This result is in accordance with a previous report that F-box protein-encoding genes showed different transcript levels during seed development, suggesting the involvement of F-box proteins in rice seed development [54].

Of the 15 known miRNA families, 8 (miR396, miR168, miR156, miR172, miR159, miR398, miR1318 and miR167) showed different levels of preferential expression in wheat flag leaves, with the logarithm of the fold changes ranged from 0.5 to 5.2 as well as more than those in the developing seeds (Figure 3a, Table 2). Putative targets of these miRNAs encode a wide range of proteins, including various transcription factors, enzymes and argonaute (AGO) protein (Additional file 11), which are involved in diverse developmental and metabolic processes. Of the 55 novel miRNAs, 28 were characterised to have different degrees of flag leaf-biased expression, with the logarithm of the fold changes ranged from 0.1 to 5.2, whereas 4 (tae-miR1120c, tae-miR1130b, tae-miR5384, and tae-miR9675) were detected only in flag leaves (Figure 3b, Additional file 5), suggesting that these novel miRNAs might participate in regulating the development and metabolism in wheat flag leaves. Fourteen of the 28 novel miRNAs with flag leaf-biased expression had putative targets, including protein kinases, glycosyltransferase, functional proteins (Histone H2B.1), and enzymes (Additional file 9), all of which are important in plant signalling pathway and metabolism. The above results imply that the regulation networks of miRNAs in wheat flag leaves are very complex. Overall, all our data suggested that wheat flag leaves have more complicated regulatory networks of miRNAs than developing seeds.

To the best of our knowledge, this study is the first to identify miRNAs with tissue-biased expression in wheat flag leaves or seeds of different developmental stage on a large scale. Sufficient wheat genome sequence data are necessary to elucidate the functions of all miRNAs preferentially expressed in wheat flag leaves or developing seeds and to reveal the involved regulatory networks.

### miRNA*s accumulation in specific wheat tissues

miRNA:miRNA* is excised by DCL1 to produce functional mature miRNA, whereas miRNA* is assumed to be gradually degraded during miRNA biogenesis [5]. However, previous studies have also suggested that miRNA*s can accumulate to a considerable level and down-regulate their target genes in both plants and animals [34,55,56]. Evidence supporting miRNA*-mediated mRNA cleavage has been recently found in *Medicagotruncatula* and maize [57,58]. In the present study, 17 miRNA*s for known mature miRNAs registered in the miRbase/*Tricum aestivum* (Release 20.0) were detected in at least one of the five libraries or tissues tested (Additional file 12). About half of these miRNA*s had relatively high tissue-biased accumulation in the developing seeds, flag leaves, or seedling. For examples, the reads of miR171b* in the 5-d, 10-d and 20-d seeds were 88, 46 and 46, respectively, which are considerably higher than those in the seedlings (1) and flag leaves (3) (Additional file 12). The highest accumulation of miR1127* was observed in the flag leaves (106 reads), followed by the 20-d seeds (53 reads). Only 1 and 2 reads were detected in the seedlings and 5-d seeds, respectively. The highest level of miR169* was observed in the seedlings (28 reads), followed by the flag leaves (5 reads). However, mature miRNA sequences for miR171b*, miR1127* and miR169* were not found in any of the five tissues tested. These results suggested that miR171b*, miR1127* and miR169* might be *de facto* miRNAs with important regulatory functions in specific tissues and developmental stages. Among the 17 miRNA*s detected, 12 had no mature miRNA sequences detected in the five libraries (Additional file 12).

The differential accumulation of miRNAs and miRNA*s in specific tissues may be attributed to the different activities of proteins involved in sRNA biogenesis. Different AGO proteins in *Arabidopsis* harbour sRNAs with different 5′-terminal nucleotides; thus, miRNAs and miRNA*s are directed in different AGO complexes [59]. A recent study revealed that different AGO-coding genes in rice have different levels of tissue-biased expression [20]. In the present study, miR168, which targets an AGO-coding gene (Ta.71657) and is the most abundant miRNA or family in the five sRNA libraries, exhibited a tissue-preferential expression, with the highest read number in the flag leaves (Table 2 and Additional file 11).

## Conclusion

Five sRNA libraries from wheat seedlings, flag leaves, 5-d, 10-d and 20-d seeds were sequenced in this study. Twenty-four known miRNAs belonging to 15 miRNA families were identified from 18 MIRNA loci in wheat, including 15 (9 MIRNA loci) first identified in wheat in the present study, 13 miRNA families (16 MIRNA loci) being highly conserved and 2 (2 MIRNA loci) moderately conserved. In addition, fifty-five novel miRNAs were also identified. The Potential target genes for 15 known miRNAs and 37 novel miRNAs were predicted using strict criteria, and these target genes are involved in a wide range of biological functions. Four of the 15 known miRNA families, including miR169, miR166, miR164 and miR160 were preferentially expressed in the developing seeds with the logarithm of the fold changes of 0.3 ~ 3.0. From 5 days post-anthesis to 20 days post-anthesis, miR164 and miR160 increased in abundance, whereas miR169 decreased, suggesting that these miRNAs have coordinating functions in the different developmental stages of wheat seed. Twenty-two of the 55 novel miRNAs also preferentially expressed in the different developmental stages of wheat seed with the logarithm of the fold change of 1.0 ~ 7.6, and half of them were seed-specific, suggesting that they participate in regulating wheat seed development and metabolism. Eight known miRNA families and 28 novel miRNAs exhibited different levels of tissue-biased expression in wheat flag leaves, with the logarithm of the fold changes ranged from 0.1 to 5.2. The potential targets of these miRNAs were involved in a wide range of biological functions, suggesting the complexity of the regulatory networks in this tissue. Our data also suggested that wheat flag leaves have more complicated regulatory networks of miRNA than developing seeds.

Sufficient wheat genome sequence annotation data are necessary to identify all miRNAs and their regulatory functions. Our dataset provides a useful source of information on miRNA regulation in wheat flag leaves and developing seeds.

## Methods

### Small RNA library construction and RNA sequencing

Winter wheat (*T. aestivum* L.) cultivar Xiaoyan 6 was used in this experiment. Wheat plants were grown under natural conditions in the experimental field of Northwest A & F University, Yangling, China (longitude 108°E, latitude 34°15′N) in 2009 to 2010. Wheat seedlings (at five-leaf stage post-vernalisation), flag leaves of heading plants and immature seeds at 5 DPA (5-d seeds), 10 DPA (10-d seeds) and 20 DPA (20-d seeds) were collected. Three independent biological replicates were included in the experiments. All tissues mentioned above were frozen in liquid nitrogen immediately after collection and then stored at −80°C until further use. Total RNA was isolated from each of these samples using TRIzol reagent (Invitrogen, Grand Island, NY, USA) according to the manufacturer’s instructions. Total RNA was extracted separately from the leaves, stems and roots of the seedlings and then mixed with equal amounts of individual parts. The integrity of the RNA samples was checked by 1% agarose gel electrophoresis. RNA samples that passed the quality check were sent to BGI (Shenzhen, China) for sRNA library construction and Solexa sequencing using standard protocols on the Illumina Hiseq 2000 platform.

### Small RNA data analysis

Small RNA libraries were constructed and sequenced for the five wheat tissues. The sequencing data were deposited in NCBI Gene Expression Omnibus (GEO, http://www.ncbi.nlm.gov.geo/) under the accession number GSE50524.

All sequencing data were first processed by filtering 5' adaptor contaminants from the 50 nt tags, removing the low-quality reads and getting rid of the sequences larger than 30 nt and sequences smaller than 18 nt using the SOAPnuke software (http://soap.genomics.org.cn/) developed by BGI, sequences with over 50% homopolymer or dinucleotide repeats were filtered out, and clean reads were generated for each sRNA library. Identical reads were subsequently pooled to create a list of non-redundant sequences (unique sequences). The unique sequences were aligned against wheat genome shotgun-sequence assemblies (http://mips.helmholtz-muenchen.de/plant/wheat/uk454survey/index.jsp) [60], and those matched to the wheat genome shotgun-sequence assemblies were kept for further analysis. Unique sequences homologous (with sequence similarity above 90%) to non-coding RNAs, including rRNAs, tRNAs, siRNAs, snRNAs and snoRNAs, were removed by BLASTN alignment against the data deposited in Rfam 10.0 (http://rfam.janelia.org/). The remaining sRNA sequences were retained for miRNA identification. To identify previously known miRNAs, these sRNA sequences were checked for an exact match (in terms of sequence and length) to a known miRNA present in miRBase (Release 20.0, http://www.mirbase.org). For those known non-wheat reference miRNAs in the miRBase, their potential miRNA precursors in wheat genome and their processing patterns (miRNA/miRNA* etc.) were further detected in the same way as novel miRNAs which was described in following section. The rest sRNA sequences were retained to predicate novel miRNAs. The potential miRNA precursors were searched by extracting the wheat EST sequences (http://www.ncbi.nlm.nih.gov/nucest/?term=wheat) or the contig sequences from wheat genome shotgun-sequence assemblies surrounding the aligned sRNA sequences, and testing their potential to form a hairpin secondary structure using RNAfold (http://rna.tbi.univie.ac.at/cgi-bin/RNAfold.cgi) [61]. The characteristic hairpin structures of miRNA precursors were used to predict novel miRNA candidates using miRNA prediction software MIREAP (http://sourceforge.net/projects/mireap/) by exploring the secondary structures, the DCL1 cleavage sites and the minimum free energy of the unannotated small RNA tags. The program was run with following parameters: Minimal miRNA sequence length (20); Maximal miRNA sequence length (23); Minimal miRNA reference sequence length (20); Maximal miRNA reference sequence length (23); Maximal copy number of miRNAs on reference (20); Maximal free energy allowed for a miRNA precursor (−18 kcal/mol); Maximal space between miRNA and miRNA* (300); Minimal base pairs of miRNA and miRNA* (16); Maximal bulge of miRNA and miRNA* (2); Maximal asymmetry of miRNA/miRNA* duplex (2); Flank sequence length of miRNA precursor (20). After this analysis, novel miRNAs have observed the following rules: (1) The set of reads from the novel miRNA locus should account for more than 95% of all the precursor mapped small RNA reads, and reliable novel miRNA reads should account for more than 75% of the corresponding set of reads; (2) the miRNA* reads should have two-nucleotide 3’ overhangs; or (3) base-pairing between the miRNA and the other arm of the hairpin, which includes the miRNA*, is extensive such that there are typically four or fewer mismatched miRNA bases, five mismatched bases being allowed if the miRNA* was detected, mean-while, no asymmetric bulges larger than two nucleotides and no more than two asymmetric bulges should be present within the miRNA/miRNA* duplex [62-64]. In addition, novel miRNAs were further identified depending on both the abundance of each sequence, which was normalized as reads per million of total miRNA reads (RPM) [65], and detection of miRNA*s. The sRNA sequences with abundance at least 5 RPM in at least one of the five tissues examined and with miRNA* detected were considered as novel miRNAs, while those had abundance at least 5 RPM in at least one of the five tissues tested but without miRNA*s detected were considered as candidate miRNAs.

### Quantification of miRNAs by qPCR

Total RNA from each of the five tissues was extracted as described above. miRNA abundance was detected according to previous reports [66,67]. Briefly, total RNA (3 μg), including miRNAs, was first polyadenylated and then reverse transcribed with poly (T) adapters into cDNA using an miRNA cDNA synthesis kit (Takara, Inc., Dalian, China) according to the manufacturer’s instructions. The cDNA products of each tissue were normalised using *UBQ* [AF517839] as the internal reference gene [68], which was confirmed to be relatively stable in the tested wheat tissues in our laboratory. These products were used as templates for qPCR. The qPCR was performed on a CFX96 Real-time System (BIO-RAD, USA) using SYBR® Premix Ex TaqTM II (TaKaRa, Dalian, China). The miRNA-specific forward primer for each miRNA was designed based on the entire miRNA sequence, and the universal reverse primer was designed based on the adapter sequence, which was provided by miRNA cDNA synthesis kit (Takara, Inc., Dalian, China) (Additional file 13). The following qPCR program was used: denaturation at 95°C for 30 s, followed by 40 cycles of 95°C for 5 s and 60°C for 30 s. Melting curve analysis with a programmed temperature ramp from 45°C to 95°C in 5 min was also performed to produce a dissociation curve for verification of amplification specificity. All reactions were run in triplicate. All primers used in this experiment are shown in Additional file 9.

The relative abundance of each miRNA was calculated by a comparative C_T_ method (ΔΔC_T_) using the formula 2^-ΔΔCT^, where ΔΔC_T=_ (C_T miRNA_ − C_T reference RNA_) − (C_T calibrator_ − C_T reference RNA_) [69]. The miRNA sample with the lowest C_T_ value that corresponds to the highest expression level was selected as the calibrator, in which the expression level was set as 1.0. The relative expression levels of the same miRNA in the other four samples were then normalised by comparing with the highest one in the tested tissues.

### miRNA target prediction

We predicted the potential targets of the newly identified miRNAs using the web-based psRNA Target program (http://plantgrn.noble.org/psRNATarget/). The following default parameters were used: Maximum expectation, 3; Length for complementarity (between the miRNA and its target) scoring, 20 [70]; Target accessibility-allowed maximum energy to unpair the target site (UPE), 25 [71]; Flanking length around target site for target accessibility analysis, 17 bp in upstream/13 bp in downstream [72]; Range of central mismatch leading to translational inhibition, 9 nt to 11 nt [73]. The custom plant transcript databases include EST database (http://www.ncbi.nlm.nih.gov/nucest/?term=wheat) and UniGene database (http://www.ncbi.nlm.nih.gov/unigene/?term=wheat) of *T.aestivum* in NCBI and contigs of *T.aestivum* [wheat gene index, release 12, http://compbio.dfci.harvard.edu/cgi-bin/tgi/gimain.pl?gudb=wheat].

## Competing interests

The authors declare that they have no competing interests.

## Authors’ contributions

HZ conceived and designed the experiments. RH, YY and CJ performed the experiments. RH, JL, YY, QC, ZL, JZ and XL analysed the data. RH, QW and HZ wrote the manuscript. All authors read and approved the final manuscript.

## Supplementary Material

Additional file 1 Summary of small RNA sequencing in the five wheat sRNA libraries or tissues. Click here for file

Additional file 2 Known miRNAs identified in five wheat sRNA libraries or tissues. Click here for file

Additional file 3 The detail information of the loci, the precursor sequences and the structures for 15 known miRNAs first identified in wheat. Click here for file

Additional file 4 The predicted pre-miRNA structures for 15 known miRNAs first identified in wheat. Click here for file

Additional file 5 The details of the 55 novel miRNAs identified in five wheat sRNA libraries or tissues. Click here for file

Additional file 6 The detail information of the loci, the corresponding precursor sequences and the structures for 55 novel miRNAs and 53 candidate miRNAs in wheat. Click here for file

Additional file 7 The predicted pre-miRNA structures for 55 novel miRNAs (tae-miR1120b~tae-miR9679) and 53 candidate miRNAs (Tae-1~Tae-53). Click here for file

Additional file 8 The details of 53 candidate miRNAs identified in five wheat sRNA libraries or tissues. Click here for file

Additional file 9 The Potential wheat targets predicted for novel miRNAs identified in five wheat tissues. Click here for file

Additional file 10 Correlation between the deep sequencing data and the quantitative real time RT-PCR (qPCR) data. Click here for file

Additional file 11 The potential targets predicted for the known miRNAs in wheat. Click here for file

Additional file 12 The abundance of the miRNA star sequences for the known wheat miRNAs detected in five wheat small RNA libraries or tissues. Click here for file

Additional file 13 The primers used in this study. Click here for file
