# Supplementary material for: Identification and characterization of microRNAs in the flag leaf and developing seed of wheat (Triticum aestivum L.)
Source: BMC Genomics. 2014 Apr 16;15:289. doi: 10.1186/1471-2164-15-289 (PMC4029127; doi:10.1186/1471-2164-15-289)
Supplement: Additional file 4 — The predicted pre-miRNA structures for 15 known miRNAs first identified in wheat. [file 1471-2164-15-289-S4.DOCX]

**Additional file 4** The predicted pre-miRNA structures for 15 known miRNAs first identified in wheat.

Note: The red letters indicate the miRNA sequences and the blue letters the miRNA* sequences. Six pairs of known miRNAs are miRNA/miRNA* in wheat).

ath-miR156a contig2137453

10 20 30 40

-- - - A U-| UGC AC

UGACAG AAGA GAGUGAGCAC CGGCG GA CGGCAUA \

ACUGUC UUCU CUCACUCGUG GCCGC CU GCCGUAU A

CG U C C UU^ --- GU

ath-miR166a , zma-miR166g-5p contig458067

10 20 30 40

-- UU G UG U A GC ACC| G

GGAAUG GUCUGGUU GAGACC GC CGCACG CG CGG CG \

CCUUAC CGGACCAG CUUUGG CG GCGUGC GC GCU GC A

CC UU G CG U G A- A--^ U

90 80 70 60 50

ccl-miR167a , bdi-miR167e contig106305

80 70 60 50

10 20 30 40

--| G - CUAAGUC C

UGAAGCU CCAGCAUGAUCUGAU GAC AUGGAU A

ACUUUGA GGUCGUACUGGACUA CUG UACCUA G

CU^ - A ------- A

70 60 50

osa-miR168a-5p,ssp-miR168a contig316572

10 20 30

C--| AU C UCA CC

UCGCUUGGUGCAG CGGGA CC GCCCG C

AGUGAACCACGUU GCCCU GG CGGGC C

CUA^ CC A C-- AG

. 60 50 40

ath-miR169b contig2537991

10 20 30

C-- UG C---| U G AG

AGCCAAGGA ACUUGCCGGCU CUGG GCU GG U

UCGGUUUCU UGAACGGCCGA GAUU CGA UU U

ACA GU UUGA^ U G GC

80 70 60 50 40

ath-miR172a , bdi-miR172a contig1109643

10 20 30 40

-- C C A ----| AUC U U

UGCAGCA CA CAAGAUUC CAUC GG CGUCG CGUAAAU \

ACGUCGU GU GUUCUAAG GUAG CC GCAGC GUAUUUA A

CU A A A UGGA^ GC- - A

90 80 70 60 50

osa-miR396d , bdi-miR396b contig4373830

10 20 30 40 50

--| CA G ACUC UUGU CUCCC UUC

UCCA GGCUUUCUUGAACUGU A GCGGGCGGA GGCG GCC U

AGGU CCGAAAGAACUUGGUA U UGUCCGCCU CCGC CGG C

AA^ AC G ---- ---- ----- CUU

100 90 80 70 60

ath-miR396b, zma-miR396f-3p contig1569374

10 20 30 40 50 60 70

-- A - - C- AAUUA -| C UCUC - U UCU

UUCCACAGCUUUCUUGA CUUC UCUUGC GCUCU CUU CC CCCCA UC UGUCUCUC CU UC C

AGGGUGUCGAAAGAACU GGAG AGAACG CGAGG GAG GG GGGGU AG ACAGAGAG GA GG C

GA G A A AC GACAG A^ A U--- A U UUG

. 140 130 120 110 100 90 80

osa-miR1432 contig2016101

10 20 30 40

A-- A A G U ---| A

UCAGG GAG UGACACCGAC CCGA CAGAUGGGU CGGCUU A

AGUCC CUC ACUGUGGUUG GGCU GUCUGCCUA GCCGGA C

ACA G C A U CGU^ C

90 80 70 60 50
