# Supplementary material for: Identification and characterization of microRNAs in the flag leaf and developing seed of wheat (Triticum aestivum L.)
Source: BMC Genomics. 2014 Apr 16;15:289. doi: 10.1186/1471-2164-15-289 (PMC4029127; doi:10.1186/1471-2164-15-289)
Supplement: Additional file 7 — The predicted pre-miRNA structures for 55 novel miRNAs (tae-miR1120b~tae-miR9679) and 53 candidate miRNAs (Tae-1~Tae-53). [file 1471-2164-15-289-S7.DOC]

Additional file 7 The predicted pre-miRNA structures for 55 novel miRNAs (tae-miR1120b ～tae-miR9679) and 53 candidate miRNAs (Tae-1～Tae-53)

The red letters indicate the mature miRNA sequences and the blue ones the miRNA star sequences

tae-miR1120b **contig3385908**

10 20 30 40

UAAA| A CUAU

UACUCCCUCUGUCCCA AAUAUAAGAACAUUUUUGACA \

AUGAGGGAGACAGGGU UUAUAUUCUUGUAAAAAUUGU U

UUUG^ G AAUG

90 80 70 60 50

tae-miR1120c **CJ629248**

10 20 30

-| A U G

CUCU UCCCA AAUAUAAGAACGUUUUUGACACUA \

GAGA AGGGU UUAUAUUCUUGCAAAAAUUGUGAU U

G^ C U G

70 60 50 40

tae-miR1122b **contig3821085**

10 20 30 40 50 60

UUA A A .-CAAA| A

UACUCCCUCUGUUCCUA AUGUAAGUCUUUUUAG GAUUUCACUG AUACAU C AUGAGGGAGGCAAGGAU UAUAUUCAGAAAAAUC CUAAAGUGAU UAUGUA G

UAA G C \ ----^ G 160 150 140 130 70 80 90

AU AUAUUU G UA U CGC

GGAC UAGA UA GAU CA \

CCUG AUCU AU CUA GU A

A- ------ - GC - UUU

120 110 100

tae-miR1122c **contig501953**

10 20 30 40

G| A A A A U

UG ACUACUCUCUCC UCCCAUAAUGU AGACGUUUUUGCAA CUA \

AC UGAUGAGGGAGG AGGGUAUUAUA UCUGCAAAAACGUU GAU A

-^ A C A C U

90 80 70 60 50

tae-miR1127b **CJ636542**

10 20 30 40 50

CAUGACA --- C ---------| C A GACUUAU

GAUGCU UCUGUCCGGAAAUACUUGUC UA GAAAU GGUGU UCUA \

UUAUGA AGGCAGGUCUUUAUGAACAG AU UUUUA CUACA AGAU U

AUA---- GGG A AUUUACCUA^ C C AUUAAUU

120 110 100 90 80 70

tae-miR1130b **contig102917**

10 20 30 40 50

AU| A U A C C U A AC A AU

UGU C CCUCC UCCCA AAUAUAAGA GUUUU GCAA CU UUG AC \

ACG G GGAGG AGGGU UUAUAUUCU CAAAA CGUU GA AAU UG C

UC^ A - C A A C C CA A UU

100 90 80 70 60

tae-miR1137b **contig920142**

10 20 30 40

UUGA| AUC C - A A

UCU UCCGUUC AGAAUAGAUGACC AACUUU UACUA A

AGG AGGCAAG UUUUAUCUAUUGG UUGAAA AUGAU A

CAUG^ A-- A G C U

80 70 60 50

tae-miR167c **contig4599680**

10 20 30 40 50 60

C - C - C C .-CUC| A AU - C

CU UUG UGG UGUGAGAGA UGAAGCUG CAGCAUGAUCUG AUCC GC GCG CCGAAC \

GA AAC ACC GCACUCUUU ACUUCGAC GUCGUACUAGAC UGGG CG CGU GGCUUG G

A C C G U - \ ---^ G -- U C

140 130 120 110 80 70

90

UAUCU AUU

UGGA U

ACCU U

U---- CGU

100

tae-miR1847 **contig2460659**

10 20 30 40

-| GC A UAACA

CCGUAACUGUACCUGCAGUUGG CAAUG CAUGUGGGCC G

GGCAUUGACGUGGAUGUCAACC GUUAC GUACACCCGG A

C^ UA C UCGGC

90 80 70 60 50

tae-miR2275 **contig941826**

10 20 30 40 50 60 70 80 90 100 110 120

AAUUUU-- A UU - U GG ACC GG -- C U UA- C .-ACACUGA| AGCUA AAG

GGCAG UGAA UGAG UGUUGGA G ACCAAAUCUU GCCG UGUGGC AAGAUUUGGUUUC UCCAA AUCUUAUGUUCA UGU AG GUGUU CAAC U

CUGUC ACUU GCUC AUAACCU C UGGUUUAGAA CGGU AUAUCG UUCUAAACCAAGG AGGUU UAGAGUGUAAGU ACG UC CACAG GUUG G

AGUAGUCC A CU U C UU CA- G- AC U - CGA C \ -------^ GUAG- AUA

260 250 240 230 220 210 200 190 180 170 140 130

tae-miR396 **contig49131**

10 20 30 40 50

-| G UC GGAUU CU UUGU C

GGCUUUCUUGAACUGU AAC GCGGGGAUGG CC GGA GGCG U

CCGAAAGAACUUGGUA UUG CGCCUCUGCC GG UCU CCGU C

C^ G UC ----- CU CUU- C

100 90 80 70 60

tae-miR397 **GH985139**

10 20 30 40

C A C A A -| C

GCA AGGUG CAUUG GUGCAGCGUUG UGAACCG UCCGGCCUC U

CGU UCCGC GUAAC CACGUCGCGGC ACUUGGC AGGCCGGAG C

- C A A C G^ C

90 80 70 60 50

tae-miR5048 **GH225205**

10 20 30 40 50 60 70 80 90 100 110 120 130 140 150 160

U C GGU GGUG U U --- U U U C U - U A -- CCCA- UC UGACUAAAAA .-AAAAAGUAAUA| GGGUG U

CUAG AAUAUAUUUGCA UUUAGGUCUAAGU AAUA CGA CUCU GAA AUA UGA AACCUU ACAAA UUGC UGAUUAUUU GUAG GCU AUUAUUGU CAUGCAUA CA AAGCA AUU UUGUG U

GAUC UUAUAUGAACGU AGAUCCAGAUUUA UUAU GUU GAGA CUU UAU AUU UUGGAA UGUUU AAUG ACUAGUAAG UAUC UGA UAGUAAUA GUACGUAU GU UUCGU UAG AACAC C

G U AC- AUUG - - ACG C U U - - A U G AA CCUAA UA ---------- \ -----------^ AAAGA U

340 330 320 310 300 290 280 270 260 250 240 230 220 170

180 190

GGAA----------- AA

AAGUUU \

UUCAAA C

AAGUAGAAGGAAGUG

tae-miR5049  **CJ725784**

10 20 30 40 50

ACGGAAGGAU| A C UA C AC

GUACUUCCUCC AUCCGUAUUA U UCUUAGAUUUGU UAGAU A

CAUGAGGGAGG UAGGUAUAAU A AGAAUUUAAACA AUUUA U

AAUAAUAGUC^ C A GC U CA

110 100 90 80 70 60

tae-miR5062  **contig4425237**

10 20 30 40 50 60 70 80

C U A - CAG-| A AC U U C A AAAACUGCUCC

GUAGCU CUUGAACCUU GG GAA CCGCAU AC CAU AACU GAAAACCA UU UCUUAUCCC U

UAUUGG GAACUUGGAA CC CUU GGCGUA UG GUG UUGA UUUUUGGU AA GGAAUGGGG A

C U - A UUUA^ C GA U - - - AAUAUAACACC

150 140 130 120 110 100 90

tae-miR5175 **CJ966226**

10 20 30 40

-| UU C AA

ACUCCCUCUGUUCCAAAUUACUCGUCGUGGUU AGU C \

UGAGGGAGGCAAGGUUUAAUGAGCAGUACCAA UCA G N

A^ AN N UU

80 70 60 50

tae-miR5384 **contig1315461**

10 20 30

U| C C C C C U

GCCGA CC UUCG CGGU GCGCGUUC CC U

UGGCU GG AAGC GCCG CGCGCGAG GG G

G^ A U U C U C

60 50 40

tae-miR6197 **contig463346**

10 20 30 40

AA| C A C U A

GUA UCCCUCUGUA ACAAAUGUAGGA GUUUU GCAGUUUA \

UAU AGGGAGACAU UGUUUACAUUCU CAAAA CGUCAAAU A

UA^ A G A U U

90 80 70 60 50

tae-miR7757 **contig491035**

10 20 30 40 50 60 70 80 90 100 110 120 130

UG CAU C CUC AGCC U- CU UUGAAACA .-AAUAUCCUUGAAC CGA CACACAU- .-ACCAAUA| AAA

CAUUA AUAAAACCUU AGCUAUCCAU CAUAUC UG CU CUUAAUU UUUUGGAAAUAUGCUUA AUAAGAA GC GCUGC CUAUG A

GUAAU UAUUUUGGAG UUGAUAGGUA GUAUAG AC GA GAAUUAG AAGAUUUUUGUAUGAAU UAUUCUU CG CGACG GAUAC G

UA AUU U AAU CUU- UC AC -------- \ ------------- C-- UCUAAUUU \ -------^ GAU

340 330 320 310 300 290 280 270 240 230 140

150 160 170 180

AAUG---- CUCUUG U U- A

AGUAAAAGAGG ACUU UAGAUCA GC A

UCAUUUUUUCC UGGA AUCUAGU CG G

AUCAUCAA CAAA-- C UU A

220 210 200 190

250

CU UA

AUAUGAU \

UGUAUUA G

C- UU

260

tae-miR9652-5p **contig176099**

10 20 30 40 50

CUA--- A C G U -- A-| UU

UUGAG UGUGUUUGUCAC UGUUU UCAUUAAGUUUCU AGUGCCUUU GCC UC \ AGCUU AUGCAAACAGUG ACAAG AGUAAUUCGAAGA UCAUGGAAA UGG AG A

AGGCGG C U - U AC GA^ UG

120 110 100 90 80 70

tae-miR9652-3p **contig176099**

10 20 30 40 50

CUA-- A C G U -- A-| UU

UUGAG UGUGUUUGUCAC UGUUU UCAUUAAGUUUCU AGUGCCUUU GCC UC \

AGCUU AUGCAAACAGUG ACAAG AGUAAUUCGAAGA UCAUGGAAA UGG AG A

GGCGG C U - U AC GA^ UG

120 110 100 90 80 70

tae-miR9653 **contig102911**

10 20 30 40

C--| C U UC - U CG

CAU GCU UGGU CCA GGCCAUGGCCAAGGUCUCG GAGGUU \

GUA CGG ACCG GGU CCGGUACCGGUUUCAGAGU UUCCAG C

GUU^ - U GU A - UG

90 80 70 60 50

tae-miR9654a **contig2975088**  10 20 30 40 50

10 20 30 40 50

U| AC GA G GGA UA

UGC GUUGGUUCGCUUCAAGCCUU GGAAUAG GUAGACUGCAU GC \

ACG CAAUUAAGCGAAGUUCGGAA UCUUAUC CAUUUGACGUG CG U

U^ AU AG A A-- UG

100

tae-miR9654b **contig4540822**

10 20 30 40 50 60

AUGCU- UUCAC-| GA G GGAGUU

CACA GUUGGUUCGCUUCAAGCCUU GGAAUAG GUAAACUGCAC \

GUGU CAAUUAAGCGAAGUUCGGAA CCUUAUC CAUUUGACGUG A

UUCUCC UACGAU^ AG A ACGUGU

120 110 100 90 80 70

tae-miR9655 **contig4369342**

10 20 30 40 50

CGGGU- C C U .-AGUG| AU

GAUCCGUCU AGGCGU GGCUACUUCC UUCCCUUGCC CGCC \

CUAGGCGGG UCUGCA CCGAUGAAGG AAGGGAACGG GCGG G

UACGAU U A - \ ----^ CC

120 110 100 90 80 60

tae-miR9656 **contig1352125**

10 20 30 40 50 60

CCA--- GCGACA A U GA- CUG-| UG

UGGCU GGCACC UUGUUCAG GUUUCGAAG AUAAUACCGUUG GGUU \

AUCGA CCGUGG GACAAGUC CAGAGCUUC UAUUAUGGCAAU UCAG G

CCCGUC AGAUA- C U GUC UUUG^ GA

120 110 100 90 80 70

tae-miR9657a **contig129692**

10 20 30

G CCG -| C GA U U GA

CGUG GCU CCGUUCG CG GAAGC UAC GCG C

GCAC CGG GGCAAGC GC CUUCG GUG CGC C

- CAG U^ U UC U C AA

70 60 50 40

tae-miR9657b **contig3619642**

10 20 30 40

AUA--- C-| ACU U GA U GA

GCGGCGUG UG CCGUUCG CG GAAGCAUGU GCG C

CGUUGCAC GC GGCAAGC GC CUUCGUGCG CGC C

CGCGCA CA^ GGU U UC C GA

90 80 70 60 50

tae-miR9657c  **contig2307022**

10 20 30 40

--| AUA AG- CUG - C GA U GGA

GC GCGG GG GCU CCGUUCG CG GAAGCAUGU GU C

CG CGUU CC CGG GGCAAGC GC CUUCGUGCG CG C

CG^ CA- GUA AG- U U UC C UGA

90 80 70 60 50

tae-miR9658 **contig543327**

10 20

U .-AACAAUAGAUU| CAG

CUGGU GGGAUU \

GACCA UCUUGA G

- \ -----------^ CGC

30

40 50 60 70 80

-- A G U UU .-GCACCUCC A

UUUG CC AUUC CCCAGAAU UGAGAAUC UAAACC A

AAAC GG UAAG GGGUCUUG ACUCUUAG AUUUGG U

AA C A U CU \ -------- A

140 130 120 110 90

A G

GAUUGU A

CUGGCA G

U A

100

tae-miR9659 **Contig972592**

10 20 30 40 50 60

U-----| AGUUGGG - A A AAC AU

CCAGGCCU GUGGAUGCUG GGA AACUAUUG ACUAGAUAAGUUAC UC \

GGUCCGGG CACCUACGGC CUU UUGGUAAC UGGUCUGUUCAAUG AG C

CCUAUC^ AAA---- A G C A-- CU

. 110 100 90 80 70

tae-miR9660 **contig102619**

10 20 30 40

- UG-| C U UUGUUAUCC

GCUCUUUCUCU CGAGCAA GGA GAAUCAGCC A

UGAGAAAGAGA GCUCGUU CCU UUUAGUCGG A

A UGA^ U C UGGAGACCU

. 80 70 60 50

tae-miR9661 **contig698108**

10 20 30

- G AG G -| CA

UUGG GAAGAUGAAGUAG CAGG ACCUCA CGCU \

AACC CUUCUACUUCAUC GUCC UGGAGU GCGA A

C A CA G G^ AG

70 60 50 40

tae-miR9662a **contig219800**

10 20 30 40

GG A -| C U AA U G AC

GG GC CCGG GGCUCUG GGUGUUCAAGCAGG CC CAU CU C

CC UG GGCC CCGAGAC CUACAAGUUCGUUC GG GUA GA G

AA G C^ A C GC C G CG

90 80 70 60 50

tae-miR9662b **contig863626**

10 20 30 40 50

UCGG A -| C CU AA UC AC

GG GC CCGG GGCUCU GGUGUUCAAGCAGG CC CAUGCU C

CC UG GGCC CCGAGA CUACAAGUUCGUUC GG GUACGA G

UAAA G C^ A CC GC C- CG

100 90 80 70 60

tae-miR9663 **contig1104456**

10 20 30 40

AA-- A CU G C C C -------| GUU

GAG AG UG UCAU UAAAG GUAGU GAACGAAUCUG CC G

CUC UC AC AGUA AUUUC CAUUA CUUGCUUAGAC GG A

ACCA A AG G C A U UUGUAUA^ AGU

100 90 80 70 60 50

tae-miR9664 **contig4244526**

10 20 30 40 50

GGUCU- UU-| C A C AUC A UG AAA

GAUGG AACGAAC ACGGCAU GAGG ACUGCAAAC CG GA AGC \

CUACC UUGUUUG UGCUGUA CUCC UGACGUUUG GC CU UCG C

UGUUAU UUU^ A G - AU- - GU AAU

110 100 90 80 70 60

tae-miR9665 **contig4097006**

10 20 30 40 50 60

-| A U C G U U GC

UAAU AU UGGUUUGA UUUGCACUGCUAGCGGUG CAC GCCGCACUGCCCGG CAGUGUAA \

AUUA UA ACUAAACU AAAUGUGACGAUCGUCAC GUG CGGCGUGACGGGCC GUCACAUU U

C^ C U C - C U AC

. 120 110 100 90 80 70

tae-miR9666a **contig4877233**

10 20 30 40

U U AU -| A CA AU A

CA UCGG UCGCCAUCAUAC GCCC ACCGUG UUUG AUGCAU U

GU AGCC AGCGGUAGUAUG CGGG UGGCAC GAGC UACGUA A

G U AU U^ A C- AC U

90 80 70 60 50

tae-miR9666b **contig5111788**

10 20 30 40

U U AU -| CA AU A

CA UCGG UCGCCAUCAUAC GUCCAACCGUG UUUG AUGCAU U

GU AGCC AGCGGUAGUAUG CGGGUUGGCAC GAGC UACGUA A

G U AU U^ C- AC U

90 80 70 60 50

tae-miR9666c **contig3367753**

10 20 30 40

AU| AU U CA AU A

UCGG UCGCCAUCAUACG CCAACCGUG UUUG AUGCAU U

AGCC AGCGGUAGUAUGU GGUUGGCAC GAGC UACGUA A

UU^ AU C C- GC U

90 80 70 60 50

tae-miR9667 **contig5124263**

10 20 30 40

-| AA U C - G

UGAAA UCUAAA AUGG AAACAAUGAAUGAGAA UCCAAAGAUA G

ACUUU GGGUUU UACC UUUGUUAUUUACUUUU AGGUUUCUAU G

U^ A- C U U U

90 80 70 60 50

tae-miR9668 **contig988741**

10 20 30 40 50 60

A C CCAA AUACAAG-- .-G| CC

GAUACAUC AUU UGACAAGUAUUUUCGGACGGAGGGA AUUC ACU A

CUAUGUAG UAA GCUGUUCAUAAAGGCCUGUCUCCCU UAAG UGA U

- U ACAG CAAAAAAAA \ -^ AG

130 120 110 100 90

70

A--- UUA

GCU C

CGA C

AUAA UCU

80

tae-miR9669  **contig288356**

10 20 30 40

G| U C U U A GAGGAG

AGA UU CAUACUG GGGCACU AUUUG CAAGAAU A

UCU AA GUAUGAU CCCGUGA UAAAC GUUCUUA A

G^ U A U - G AGUACA

80 70 60 50

tae-miR9670 **contig862671**

10 20 30

-| C C UC

CCUGAA GUCUUCUUCAAGUA UCCACUUUUUUUUC \

GGACUU UAGAAGAAGUUCAU AGGUGGAAAAAGAG G

U^ U A GC

70 60 50 40

tae-miR9671  **contig24491**

10 20 30 40

-| A CAA C UGAUA

UAUUGUGAU UGACUUUACA CUGUC GGCAGUACAUUGUC G

AUGACACUA ACUGAAAUGU GACGG UCGUCAUGUGACAG U

U^ A AUG C UCAUG

90 80 70 60 50

tae-miR9672 **contig3459861**

10 20 30 40 50 60

A- G A A - .-AAGA| G ACUA

UCU UUUGA GUGAAG UGAUGCUUAAUGACAGUCGUGGU UCUUAG GCCAG CG U

GGG AAACU CACUUC GCUACGAAUUACUGUCAGCACCA AGGAUC CGGUC GC G

CC A C C C \ ----^ - AUCU

140 130 120 110 100 70

80

CAUUAA UC

GCA G

CGU U

------ UC

90

tae-miR9673 **contig477975**

10 20 30 40

- C - C U GGAG---| AAGA

CG CUCGA GGUAAGAAG AAA AGCACAUGCA GU \

GC GAGCU CCAUUCUUC UUU UCGUGUGCGU CG A

G C C C C AAGCGAA^ AAGA

. 80 70 60 50

tae-miR9674a **contig2673750**

10 20 30 40

AAU U AUUCAAC-| U

UUGUCCAUAGCAUCA CCAUCCUACC GGCGGC G

GAUAGGUAUCGUGGU GGUAGGAUGG CCGUCG C

UUC C GUGGCCAC^ U

80 70 60 50

tae-miR9674b **contig3591082**

10 20 30 40

-| CUC C U C UUCA G UUGCC

GAA GU CAUAGCAUCA CCAUC UACCC AC GUGG \

CUU UA GUAUCGUAGU GGUAG AUGGG UG CACC G

A^ CAA A C A ---- G CCUUA

90 80 70 60 50

tae-miR9675 **contig824138**

10 20 30 40 50

A C C C C A | AC

UG UG CAG CAAGACGAGGGUGAUCAUAAACU CUGGGU AUCAUG--UUGGAC \

AC AC GUC GUUUUGCUCUCACUAGUAUUUGA GACCCG UAGUAU GACCUG C

- U U C C G \ ^ AC

220 210 200 190 60

70 80 90 100

.-GAAC A- GA GAGA A

AUGCCG GGAGUUCA GCG GGUUC A

UGUGGU UUUCAGGU CGU UCGAG G

\ ---- AC A- ---- G

120 110

130 140 150

AUU----- C CAGA A

CUUCGGGUACA GCCG CC \

GAAGUCCAUGU CGGU GG A

UUGAGGAC A A--- A

180 170 160

tae-miR9676 **contig692628**

10 20 30 40 50 60

CU| A U - U CCC AUCCA U UUC

UCAC CCGUGGAUGUCAUCG GGCCGUACA UGAA CGUCC AC GC CUG A

GGUG GGCACCUACAGUAGU CCGGCAUGU ACUU GCAGG UG CG GAC A

UC^ C - U U --- AAA-- - UUU

110 100 90 80 70

tae-miR9677 **CJ576106**

10 20 30 40 50

CAA--| C UC C C A UCCCCC

AGG GGCC GCUU UCUUC ACUCUACCAAC GCCACGUC G

UCC CCGG CGAG AGAGG UGAGAUGGUUG CGGUGCGG C

CCUUG^ A UA C A C UACCAG

100 90 80 70 60

tae-miR9678  **CJ652184**

10 20 30 40 50

-| G A G A C A GAAU CG

CGUAG AUC UCUG CG GGGACAUA ACUGUACA GU CGCGG C

GCAUC UAG AGAC GC CCCUGUAU UGACAUGU CG GCGUC G

A^ - C G - U C ---- CC

90 80 70 60

tae-miR9679 **contig3874161**

10 20 30 40

-| C C AU CC CA

GAGGCGC UC AGAACCAGAAUGAGUAGCUC GCA UACU A

CUUCGCG AG UUUUGGUCUUACUCGUCGAG UGU GUGA C

C^ U A -- AA UA

80 70 60 50

Tae-1 **contig558785**

10 20 30 40

A CAAAU GA .-A| U

AUCUUU UUUUUUGUU ACAUAAAUAU UAAA A

UAGAAA AAAAAACAG UGUAUUUAUG AUUU C

- AUCUU GC \ -^ G

100 90 80 70

Tae-2  **contig2220558**

10 20 30 40 50 60 70

CUUUA --- UAAA CUACAUCU- A C--| C CA

CAAACUUU GACCAAGUUUGUAGAGA UAU AGAAUACCA CUA AUAAU AUGAGA \

GUUUGAAA UUGGUUCAAAUAUCUCU AUA UUUUAUGGU GAU UAUUA UACUUU U

CUUCA UGU UUCG AUAUACAUU C AUA^ - UA

150 140 130 120 110 100 90 80 Tae-22

Tae-3  **contig94033**

10 20 30 40

A -------| UC UUUU

UCCUCU--GGUUGAAUUUGUCCAUAGCAUCAGCCA AUCC UGU \

AGGAGA CCAACUUAAAUAGGUAUCGUGGUCGGU UGGG GCA U

A \ AGCAUCA^ UA UGUU

120 90 80 70 60 50

Tae-4  **contig3481471**

10 20 30 40

GG| G U CU G U CA AC AU

CU CCACU AAUCAC U CGGC UGA CCGC UG \

GG GGUGG UUGGUG A GUCG GCU GGUG AC G

GA^ A - -- G U -- CA AG

80 70 60 50

Tae-5  **contig20256**

10 20 30 40 50

-| C A U U CACC A C UGA

CUG CGG CGUUUUU GAUCC UAGGAUGGCCCGA UG GCC GAGC C

GGC GCC GCAAGAA CUAGG AUCCUACCGGGCU GC CGG CUCG C

C^ A C - U UCU- A A CCC

100 90 80 70 60

Tae-6  **contig567161**

10 20 30 40 50 60 70 80 90 100 110

U C A A U AC U A CAA .-AAA| G

GUU CAUAAU UAGUGCCUAUA AUUUUUUA AAAGUCAAACUUUAUAAACUUUGACCAAGUUUAU AGAAAA UAUUUAUAU UA UGUUAAAUAUAUA UAU A

CAA GUAUUA AUCACGGAUGU UAAAAAAU UUUCAGUUUGGAAUGUUUGAAACUGGUUCAAAUG UCUUUU GUAAAUGUA AU ACGGUUUGUAUAU AUA A

G - C C U CC C A CUC \ ---^ A

240 230 220 210 200 190 180 170 160 150 140

120

---------- U

CAUC U

GUAG A

AAUAAUCUAA C

130

Tae-7  **contig134713**

10 20 30 40 50

C C A C U CCA---| C

GGUU UU GUCUAAAUCAUCCCAUCCUCAA AU CUUUCACAG UGGC \

UCGA AA CAGAUUUAGUAGGGUAGGAGUU UA GAAAGUGUU AUCG A

C A A A U AUAUAC^ A

100 90 80 70 60

Tae-8 **contig219800**

10 20 30 40

GG A -| C U AA U G AC

GG GC CCGG GGCUCUG GGUGUUCAAGCAGG CC CAU CU C

CC UG GGCC CCGAGAC CUACAAGUUCGUUC GG GUA GA G

AA G C^ A C GC C G CG

90 80 70 60 50

Tae-9 **contig2591194**

10 20 30 40 50 60 70

UU U A C - A C UG C---| U G AG

GCCA ACGCU AGAGG AGAGAAC GGG UG AGCCAAGGA ACUUGCCGGCU CUGG GUU GG U

CGGU UGUGG UCUUC UCUCUUG UCC AC UCGGUUCCU UGAACGGCCGA GAUU CGA UC U

UC U - U A - A GU UUGA^ U G AC

140 130 120 110 100 90 80

Tae-10  **contig768867**

10 20 30 40 50 60 70

GU AA A AGCGUU-- -| A G CAU AG

UGCUACCAUGAAGAGCGCGGGCAGCACAACCG GGC CU GCGUA GG GG GCUG CGC \

GCGAUGGUACUUCUCGCGCCCGUCGUGUUGGC CUG GA CGCGU CC CC CGGC GCG C

CU -- C CCAUACUU G^ A G U-- UA

140 130 120 110 100 90 80

Tae-11  **contig849921**

10 20 30 40

U A C .-UUUAU| AGC

AGCUGCCA AAUCCA UUCUAUCAGGUUCUUGAG AGAAG U

UUGAUGGU UUAGGU GAGAUAGUCCAGGAACUC UCUUC G

- G A \ -----^ AAU

130 120 110 50

60 70

CUU U

UUCU--GCCC \

AAGA CGGG G

U-- \ U

100

80

U--- UUGC

GACC G

CUGG C

UUCU UAAA

90

Tae-12  **contig1266755**

10 20 30 40

CCAACC UG C - AU- | CU

CUAGUAUG GU UAC UAUGCCA GAUGGCU--GG \

GGUCAUAC CA AUG AUACGGU CUACCGG CC G

AUACAU GU C A AUC \ ^ UC

260 250 240 230

50 60 70 80 90 100 110 120 130

UC- C ACAUAC C A .-AUCGUG A U UACA --- UG- A

UCCUAGGC UACUUGCAU AUUAUCG CAUCCAGG AACUCC UGGGA CCA GCA AUUGCCA CCA UC C

AGGGUCUG AUGGAUGUG UAGUAGU GUAGGUUC UUGAGG GCCCU GGU CGU UAACGGU GGU AG U

GCA C C----- A C \ ------ - - CUA- ACG CAA A

220 210 200 190 160 150 140

170

AC G

GUAC U

CAUG A

C- C

180

Tae-13  **GH225472**

10 20 30 40 50 60 70

UA CA A C U AGU C- ------| AAAAU AU

UUACUC UCC UUCCUAAAUAUAAGUCUUU UAGAGAUU CACUA GACUACAUA GA AGC GA \

GAUGAG AGG AAGGAUUUAUGUUCAGAAA AUUUCUAA GUGAU CUGAUGUAU CU UCG CU G

UA AG C A U CCC AC ACAUUC^ CAU-- AA

150 140 130 120 110 100 90 80

Tae-14  **contig916294**

10 20 30 40

UG UA--------| C C CAA

GUAC CCUCUGUUCACUAAUGUAGGA GUUUU GCAGUU \

CAUG GGAGACAAGUGAUUACAUCCU CAAAA CGUCAA U

UA UGUUGAUAUG^ U A AUU

90 80 70 60 50

Tae-15  **contig05941**

10 20 30 40 50 60 70

- U G C UG U U UC A .-C| UC

GUGGCGGUCC CAC AGUUCUUGAGUCUGCA UUGUUUU GC UGUA CUCUUGUCAACGC CCG CCUA CAG U

CAUCGCCAGG GUG UUAAGGACUCGGACGU GGCGAAG CG ACGU GAGAACAGUUGUG GGC GGAU GUC G

C - G C GA - U U- - \ -^ CU

170 160 150 140 130 120 110 80

90

A- UC

GAAAA A

CUUUU U

UU UG

100

Tae-16  **CJ862830**

10 20 30 40

CC UC -| UG

CCC CG AUCCAAAAUAAGUGUCGCGGUUUUGAACUAAGGUUG G

GGG GC UAGGUUUUAUUCACAGCGUCGAAACUUGAUUCCAAC G

GA UU U^ UU

90 80 70 60 50

Tae-17 **contig79179**

10 20 30 40

UUAUAUA| A A A

CUCCCUCCGUCCCACA UAUAAGACGUCUUUG AAGCUA \

GAGGGAGGCAGGGUGU AUAUUCUGUAGAAAC UUCGAU C

UUAUUAC^ C G A

90 80 70 60 50

Tae-18  **CJ723354**

10 20 30 40 50

AUGCC| A G C A GA

UACUCCCUCUGUUCC AAGUAA UGUCG GGUUUUAGUUCAA UUU A

AUGAGGGAGGCAAGG UUUAUU ACAGC CCAAAAUCAAGUU AAA C

AGAAC^ C A A C AU

100 90 80 70 60

Tae-19  **contig28919**

10 20 30 40

U| CUA UUA CUA

AUUCCCU UUCGGAAUUACUUGUCUCAGAAU GUU U

UAAGGGA AAGCCUUAAUGAACAGAGUCUUA CAA A

-^ AAC C-- CAU

80 70 60 50

Tae-20  **contig03265**

10 20 30 40

UUAAG A U A CUAG -| UUC

CACU AAGCCUUAG GA UAUCUACA UAAG AUUUUC A

GUGG UUCGGAAUC CU AUAGAUGU AUUC UAAAGG U

UUAUG G C C UACG G^ UUG

90 80 70 60 50

Tae-21  **contig00494**

10 20 30

AAA| C C C C A C

AGUA UC CUCU UCC AUAAUAUAAGAG CG \

UUAU AG GAGG AGG UAUUAUAUUCUC GC U

UCA^ A A C U - A

70 60 50 40

Tae-21 **contig68480**

10 20 30 40

AAUCU| C C A AG A U

UACUC CUCCGUCC AUAAUAUAA AUC UUUGC AGUUG \

AUGAG GAGGCAGG UAUUAUAUU UAG AAACG UCGAU U

ACUAC^ A U C CA C U

90 80 70 60 50

Tae-22  **contig389178**

10 20 30 40 50

UAAUAAU| U G

UACUCCCUCUGAUCUA AGUAAGUGUUGCGGUUUUGAAUUAAGGUU A

AUGGGGGAGACUAGGU UUAUUCACAGCGCUAAAACUUGAUUCCAA A

AUACUCC^ U U

. 100 90 80 70 60

Tae-22 **contig1396484**

10 20 30 40 50

AAUCUAA| CA G A

UACUCCCUCUGAUCCAAAAUAAGUGU UGGUUUUAG UUA A

AUGGGGGAGACUAGGUUUUAUUCACA ACCAAAAUC AGU U

UUUUGUC^ AC A U

. 90 80 70 60

Tae-23  **contig03265**

10 20 30 40 50

UAAA| AG UGCA CUUG UUU UCA

GC UCGAGCCUUAG UAUCUACAC AAGU UUUCU \

UG GGUUCGGGAUC AUAGAUGUG UUCA AAAGA U

GUUA^ GU CCUC ACAA U-- CCG

. 90 80 70 60

Tae-24  **contig1916243**

10 20 30 40 50

UAA| UUC - AA

GGCAAUUUUAGUUGACGCGAGGAUGACAAGUUUAGCUGGCUU CA GA A

CCGUUAAAAUCAACUGCGUUCCUAUUGUUCAAAUCGACCGAA GU CU U

CUA^ C-- A GU

100 90 80 70 60

Tae-25  **contig4824289**

10 20 30

---------| A A - AAG C

GG CGUUCUUCUCCCUC UCCUCCU CU AC U

CC GCAAGAAGGGGGAG AGGAGGA GA UG U

CAGCCACUA^ A - C AA- G

70 60 50 40

Tae-25 **contig1025429**

10 20 30 40

U| UCUUAG GA CA CAAAG C

GUUG GG CGUUCUUCUCCU UCCUCCU AC U

CAGC CC GCAAGAAGGGGG AGGAGGA UG U

-^ CACUA- A- AG CGAAA G

80 70 60 50

Tae-26  **contig83006**

10 20 30 40 50 60

-| A C C AUA AGCA UAUGCUUUUG

UCAUGAAAGA CAUU GUGGAAGU GCGGCAAAAA ACAAAAUC CUCCAA \

AGUACUUUCU GUAA CACCUUCA CGCCGUUUUU UGUUUUAG GAGGUU A

U^ A A A A-- CUAC UUCACUACAA

130 120 110 100 90 80 70

Tae-27  **contig714600**

10 20 30 40 50 60 70

- AA- A A AU AU - --| A AAAAUAACA AG- AG CAUC

GGU AG AGGAGG GAAG GAAGGACGACC C AC CA CAGUC GUC UGAGUG UGUA \

CCA UC UCCUCC CUUC CUUCUUGCUGG G UG GU GUCGG UAG GUUCAC ACAU A

U GAG - C CU GG A CU^ C CCAAG---- AGA CA AUAU

150 140 130 120 110 100 90 80

Tae-27  **contig1969522**

10 20 30 40

U GA GG U --| AG U

GUCG G GGUCGUCCUUC UCUUCUCUUCCU CUU AC U

CGGC C CCAGCAGGAAG AGAAGAGGAGGA GAA UG U

- UA UA U AC^ A- G

80 70 60 50

Tae-28  **contig943666**

10 20 30 40 50 60

G G -| UU CACU GU- CCUA

ACGAUACU UUGGACGAUUCAGAGACGAUA UGU AAUC GGCUGU UGCA G

UGCUAUGA AACCUGCUAAGUCUCUGCUAU ACG UUAG CUGGUA AUGU U

G G C^ U- U--- GAU UUCC

. 110 100 90 80 70

Tae-29  **contig385694**

10 20 30 40 50

AUA| C C C UA- AUA C UAUAU

CUC UC GUUUCGAAUUACUUG CGCAGA UGA UAU UAGA \

GAG AG CAAGGUUUAAUGAGC GUGUCU ACU AUA AUCU U

UUA^ - A A UUA AC- A UGAUU

100 90 80 70 60

Tae-30  **contig496472**

10 20 30 40

UG| C U UA

UACUCCCUCCGUUC GAUUUACUUGUCGUGGUUU AGUU A

AUGAGGGAGGCAAG CUAAAUGAGCAGUACCAAA UCAA A

UG^ A - UU

80 70 60 50

Tae-30 **contig500041**

10 20 30 40

ACU| C C A

ACUC CUCCGUUCUGAU UACUCGUCAUGGUUUUAGUUCA A

UGAG GAGGCAAGACUA AUGAGCAGUACCAGAAUCAAGU U

UUU^ A A U

. 80 70 60 50

Tae-31  **contig08917**

10 20 30 40 50

-| CG C UAU UAG

CCUCCGUC AAAUACUUGUCGGA AAAUGGAUG GUAGAUGUAUUU \

GGAGGCAG UUUAUGAACAGCUU UUUACUUAU UAUCUACAUAGA U

G^ AG C UUU UGU

100 90 80 70 60

Tae-32  **contig493751**

10 20 30 40 50

U| G CGC AAAACA CUAA

UCUACUUCCUC GUUCCAAAUUACU CGCA AAUGUAUCUGAA A

AGAUGAAGGAG CAAGGUUUAAUGA GUGU UUACAUAGAUUU A

U^ G ACA CCAUAC ACAU

110 100 90 80 70 60

Tae-32 **contig111099**

10 20 30

UACUACUCC G -| GA

UUUCGUUCUAAA UACUUG UAA C

GAGGCAAGGUUU AUGAAC GUU A

UUUAUAUUA A A^ AA

60 50 40

Tae-33  **contig3049721**

10 20 30 40 50

AUA| U C UCUA C CAG

GCUACUCCCUCUGAUCCAUA UACUUGUCG UCA GA GUAUUU \

UGAUGAGGGAGGCUAGGUAU AUGAACAGC AGU CU CAUAGA U

AUA^ C A UUAC A UCG

100 90 80 70 60

Tae-34  **contig347151**

10 20 30 40

GUACGUA| C U C

CUCC UCUGUCC GUAAUAUAAGAGCGUUUUUGACAUUA \

GAGG AGGCAGG UAUUAUAUUCUCGUGAAAAUUGUGAU A

AAUUUAC^ U U C

90 80 70 60 50

Tae-35 **contig60545**

10 20 30 40 50

- C - CA A--| AU AAAUCCUAAUCC

CG CUCGA GGUAAGAAG AAGAGCACAUGCAUUCA CUG CU \

GC GAGCU CCAUUCUUC UUCUCGUGUGCGUAAGU GAC GA C

A C C C- AAA^ CC CUACUCUUCACC

110 100 90 80 70 60

Tae-35 **contig1031547**

10 20 30 40 50

- C - C A-------| CU UCCUAU

CG CUCGA GGUAAGAAG AAAGAGCACAUGCAUUUA CUGAU GAA \

GC GAGCU CCAUUCUUC UUUCUCGUGUGCGUAAGU GACUA CUU C

A C C C AAAAACCC^ CU CACCCC

110 100 90 80 70 60

Tae-36  **contig61229**

10 20 30

AU| C C A A

UACU CCUCCGUUC AAAUUACUC UCGUGGUUUUG \

AUGA GGAGGCAAG UUUAAUGAG AGCACCAAAAU G

UU^ A A C C

70 60 50

Tae-36 **contig1659986**

10 20 30 40

UCG C -| A

UACUCCCUCCGUUCUAAAUUACUCG CGUGG UUUAGUUCA U

AUGAGGGAGGCAAGAUUUAAUGAGC GCACC AAAUCAAGU U

GG- A A^ U

. 80 70 60 50

Tae-37 **GR503881**

10 20 30 40 50 60 70

CUUUUGCCG GU CC U C UC C .-UGAUCGA| A AGA ACUA

GU UUGUCC AU CAGUAGA GA--UGU CGA GAUG UG CAC UGAAGA \

CA AACAGG UA GUCAUUU CU ACA GCU UUAC AC GUG ACUUCU U

UCUACUUAA AC AU C U \ GA - \ -------^ C AAA AUCA

180 170 160 150 120 90 80

100

G CAAA

GCAAG \

CGUUC A

U CCCC

110

130 140

AUCCCACUCCCCC U

GC C

CG C

CU----------- U

Tae-38 **contig1941326**

10 20 30 40 50

U-- - U C UG U C - C U-| UA U

CC UCUGGUU GA U UCCAUAGCAUCA CCAU CUG CCAUU UGC GAGG GC G

GG AGACCAG UU A AGGUAUCGUGGU GGUA GAU GGUAG ACG UUCC CG C

AUC U C A GU C A G C UU^ -- U

110 100 90 80 70 60

Tae-39  **contig1941326**

10 20 30 40 50

U-- - U C UG U C - C U-| UA U

CC UCUGGUU GA U UCCAUAGCAUCA CCAU CUG CCAUU UGC GAGG GC G

GG AGACCAG UU A AGGUAUCGUGGU GGUA GAU GGUAG ACG UUCC CG C

AUC U C A GU C A G C UU^ -- U

110 100 90 80 70 60

Tae-40  **contig951499**

10 20 30 40 50 60 70

U| A U C ACUAUCAAUA UC U G

GACCAACUUUAUAGGAAA AGUAGUAGCAU UAUGGCA UAAAUU GGU AUU UGAAAU \

CUGGUUGAAAUAUCUUUU UUAUCAUCGUA AUACUGU GUUUAA CCA UAA ACUUUG U

C^ C U A ---------- UA U A

130 120 110 100 90 80

Tae-41  **HX072167**

10 20 30 40 50

CAGAGGAAGAC C UU C AU U A-| U

GA GUCGCCGGCGAGCGCC UCG CGGA C CGUGC GC C

CU CAGUGGCUGCUCGUGG AGC GCCU G GCGCG CG U

GCAGCGU---- A UU U CG C GC^ G

100 90 80 70 60

Tae-42  **contig1522795**

10 20 30 40 50 60 70

UGAUC-| CU A U - U C ACAUCCAGC UC

GGGAG UCAC CCGUGGAUGUCAUCG GGCCGUAUA UGAA CGUCC CC UCUGA A

CCCUC GGUG GGCACCUACAGUAGU CCGGCAUGU ACUU GCAGG GG AGACU A

ACAAGA^ UC C - U U U ACAU----- UU

. 130 120 110 100 90 80

Tae-43 **contig121009**

10 20 30 40 50

UGAU ACC C -| U UUCA

GGU CUUCGACCCU UUUAGUUCAACUAAAUGAA UAAA UUUCAGAA C

CCA GAGGCUGGGA GAAUCAAGUUGGUUUACUU AUUU AAAGUUUU U

ACGC GAC U G^ C UACU

110 100 90 80 70 60

Tae-43 **contig12820**

10 20 30 40 50

UU| UU U C AUGAAA CA

GAGGU CCCUCUGACCCUAUUU GUUCAA CAAAUGAA UUUUAGAAUU \

UUCCA GGGAGGCUGGGAUGAA CAAGUU GUUUACUU AAGGUUUUAA C

AU^ U- U A G----- UU

110 100 90 80 70

Tae-44  **contig4164108**

10 20 30 40

- A CGA -| AGGA UC

AGUUAG GUUUGGA GGAUGUGCAGCUG CGGU CGU A

UCGAUC CGAACCU CCUACACGUCGAC GCCA GCA G

A G UCA G^ ---- UC

80 70 60 50

Tae-44 **contig254772**

10 20 30 40

- A - A - A--| ACU

UAGUUAG G UUGGACG GGAUGUGCAGCUG CGGU GG C

AUCGAUC C AACCUGC CCUACACGUCGAC GCCA UC U

G G G A G GCA^ GAC

80 70 60 50

Tae-45 **contig255044**

10 20 30 40

UAUU C C C -- ----| C

AG UACUCC UC GUCUCAUAAUAUAAA CGUUUUU UACA U

UC AUGAGG AG CAGGGUAUUAUAUUU GCAAAAA AUGU A

AGUC U A A UC CUUG^ G

90 80 70 60 50

Tae-45 **contig3218027**

10 20 30 40

UUACAUUCUA| C C GA G

UCC UC GUCCUAUAAUAUAGGAUGUUUUU ACACUA \

AGG AG CAGGGUAUUAUAUUCUGCAAAAG UGUGAU U

UCAUUAUAUG^ A A AC G

90 80 70 60

Tae-46  **contig4075038**

10 20 30 40

ACUUG U ---| G A

UUAC ACCUCUGUCCCAAAAUAUAAGACGU UGCAG UCA A

GAUG UGGAGGCAGGGUUUUAUAUUCUGCA ACGUU AGU U

CACUU U AAA^ A U

90 80 70 60 50

Tae-46 **contig1116676**

10 20 30 40

ACUUG| U G G A

UUAC ACCUCUGUCCCAAAAUAUAAGACGUU UUGCAG UCA A

GAUG UGGAGGCAGGGUUUUAUAUUCUGCAA AACGUC AGU U

CACUU^ U A A U

90 80 70 60 50

Tae-47  **contig94531**

10 20 30 40

CU -| C G UCU GU

AGUGGAGGAUUCCA AGGGAU GCAUUGAUC AUC CC G

UCGCCUCUUAAGGU UCCCUA CGUGACUAG UAG GG C

GU C^ A G CU- AA

80 70 60 50

Tae-48 **contig444356**

10 20 30 40 50

GCAUUAAAUC| G UAAA AG UU UCA

ACUU AGCCUUAG UAUCUACACU UAAGU UUUCU \

UGGA UCGGAAUC AUAGAUGUGA AUUCA AAAGA U

GUUGUU----^ - CCUC CG U- UUG

100 90 80 70 60

Tae-48 **contig2067906**

10 20 30 40 50

----| A UU AG AAU U A CUAG U UUC

CAA GC UA CACU UAAGCCUUAG GA UAUCUACA UAAGU UUUC A

GUU CG AU GUGG GUUCGGAAUC CU AUAGAUGU AUUCG AAAG U

GUGU^ G UU G- --- C C UACG U UUG

11

Tae-49 **contig620461**

10 20 30 40 50

-| U U U UA A UAU

CUCUCUCU UUUGGAAUUACUUGUC CGGA AUGGA UAUCUAGA C A

GAGGGAGG AAGCCUUAAUGAACAG GCCU UACCU AUAGAUCU G A

U^ U C U GC - UAU

100 90 80 70 60

Tae-50  **contig2441482**

10 20 30 40

-| CAGG A C C A GC

UGC GGCGGC CUG CGUACUGUC GG CAGUGUAA \

ACG CCGUCG GAC GCGUGACGG CC GUCACAUU U

G^ UUGA A U U C AC

80 70 60 50

Tae-51 **contig2963542**

10 20 30 40 50

C| AC G GAU G GGA UA

UGC GUUGGUUCGCUUCAAG CUU GAAUAG GUAAACUGCAC GC \

ACG CAAUUAAGCGAAGUUU GAA CUUAUC CAUUUGACGUG CG U

U^ AU G AGU A A-- UG

100 90 80 70 60

Tae-52  **contig1307888**

10 20 30 40 50 60

A| ACCUACAC A U GA ---- G G GA

AUGCUC GUUGGUU GCUUC AGCCUU GGAAUAG GG UA ACUGCACA G

UACGAG CAAUUAA CGAAG UCGGAA UCUUAUC UU AU UGACGUGU C

-^ -------- A U AG AUAG G G AU

110 100 90 80 70

Tae-53  **contig230466**

10 20 30 40 50

U| CCCGCA CUC A G AAUCU

GCUA CAGAUCCUUUUGG UGGUAGCAAUUCCA CC UAC G

CGAU GUUUAGGAAGACC GCCAUCGUUAGGGU GG GUG G

A^ AAACAC UUA C G CACAU

100 90 80 70 60
