# Supplementary material for: Identification and characterization of microRNAs in the flag leaf and developing seed of wheat (Triticum aestivum L.)
Source: BMC Genomics. 2014 Apr 16;15:289. doi: 10.1186/1471-2164-15-289 (PMC4029127; doi:10.1186/1471-2164-15-289)
Supplement: Additional file 10 — Correlation between the deep sequencing data and the quantitative real time RT-PCR (qPCR) data. [file 1471-2164-15-289-S10.DOCX]

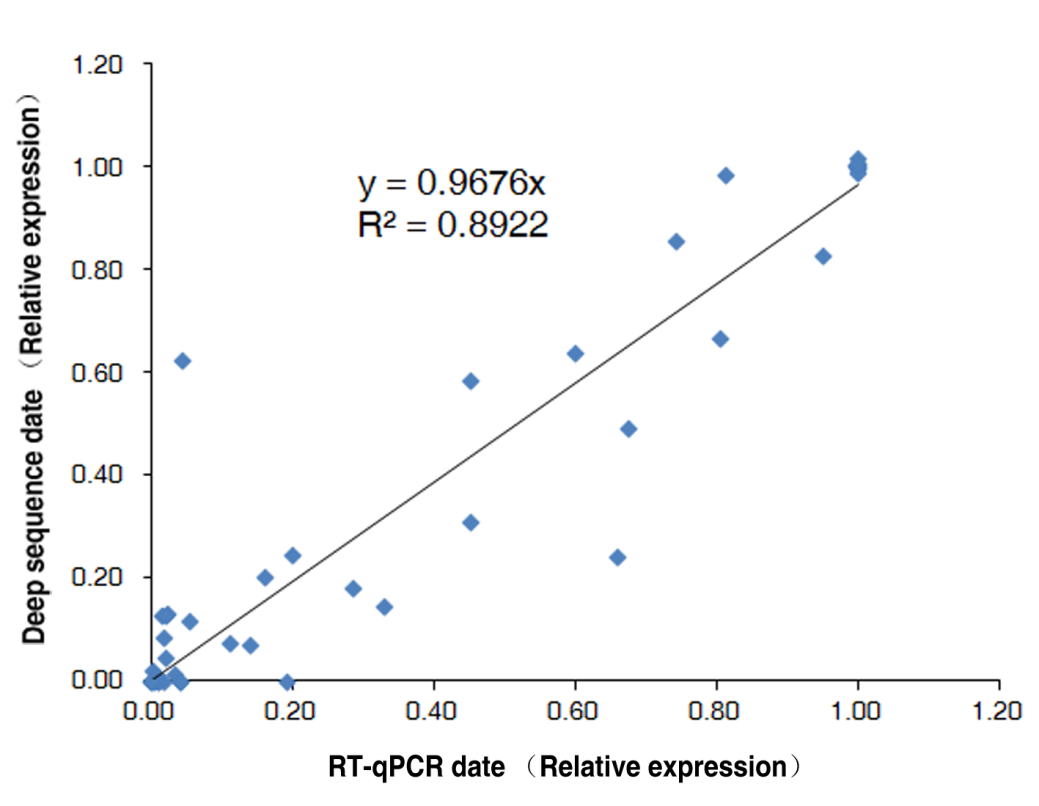


**Additional file 10** [**Correlation**](app:ds:correlation) **between** **the deep sequencing data and the quantitative real time RT-PCR (RT-qPCR) data**. The PCR templates were normalized with *UBQ* (AF517839) as a internal reference. In RT-qPCR method, relative expression of each miRNA was caculated by a comparative C_T_ (ΔΔC_T_) method ([Livak and Schmittgen, 2001](#_ENREF_31)). The miRNA sample with the lowest C_T_ value, and thus the highest expression level, was selected as the calibrator, in which the expression level represents 1.0,and the relative expression of the same miRNA in other four samples were then normalized by comparing to the highest one in the tested tissues. Three independent biological replicates were performed in this experiment. For each sample, RT-qPCR was operated in triplicate. In deep sequencing technology, read counts for each miRNA in one sample were normalized to reads per million of total reads (RPM), The relative expression of each miRNA was calculated by setting the highest read number (RPM) of each miRNA across the five samples as 100%, the relative expression of the same miRNA in other four samples was its RPM divided by the highest one.
