# Supplementary material for: Identification and characterization of microRNAs in the flag leaf and developing seed of wheat (Triticum aestivum L.)
Source: BMC Genomics. 2014 Apr 16;15:289. doi: 10.1186/1471-2164-15-289 (PMC4029127; doi:10.1186/1471-2164-15-289)
Supplement: Additional file 13 — The primers used in this study. [file 1471-2164-15-289-S13.DOCX]

Additional file 13 The primers used in the present study

| **Name** | **Sequence(5’-3’)** |
| --- | --- |
| miR169 forward primer | CAGCCAAGGATGACTTGCC |
| miR164 forward primer | TGGAGAAGCAGGGCACGTGCA |
| miR172 forward primer | AGAATCTTGATGATGCTGCAT |
| miR396 forward primer | TCCACAGGCTTTCTTGAACTG |
| miR159 forward primer | TTTGGATTGAAGGGAGCTCTG |
| tae-miR1127b forward primer | ACAAGTATTTCTGGACGGAGG |
| tae-miR397 forward primer | TCACCGGCGCTGCACACAATG |
| tae-miR5049 forward primer | GGAATATGGATCGGAGGGAGTAC |
| Tae-13 forward primer | GCAGACTTGTATTTAGGAACGGA |
| **Universal reverse primer*** |  |
| UBQ forward primer | TTTTGTAAAGACCCTCACCG |
| UBQ reverse primer | GATGCCCTCCTTGTCCTG |

***** Universal reverse primer was designed based on the adapter sequence, which was provided by the miRNA cDNA synthesis kit (Takara, Inc., Dalian, China).
